# COVID-19 and Cerebrovascular Diseases: A Systematic Review and Perspectives for Stroke Management

**DOI:** 10.3389/fneur.2020.574694

**Published:** 2020-11-05

**Authors:** Pedro Fraiman, Clecio Godeiro Junior, Elena Moro, Francesco Cavallieri, Marialuisa Zedde

**Affiliations:** ^1^Division of Neurology, Hospital Universitario Onofre Lopes, Universidade Federal do Rio Grande do Norte, Natal, Brazil; ^2^Division of Neurology, Centre Hospitalier Universitaire of Grenoble, Grenoble Institut of Neuroscience, Grenoble Alpes University, Grenoble, France; ^3^Neurology Unit, Neuromotor & Rehabilitation Department, Azienda Unitá Sanitaria Locale - Istituto di Ricovero e Cura a Carattere Scientifico di Reggio Emilia, Reggio Emilia, Italy; ^4^Clinical and Experimental Medicine PhD Program, University of Modena and Reggio Emilia, Modena, Italy

**Keywords:** COVID-19, coronavirus, cerebrovascular, intracranial hemorrhage, SARS-CoV-2, stroke

## Abstract

**Importance:** Reported cerebrovascular events in patients with COVID-19 are mainly ischemic, but hemorrhagic strokes and cerebral venous sinus thrombosis (CSVT), especially in critically ill patients, have also been described. To date, it is still not clear whether cerebrovascular manifestations are caused by direct viral action or indirect action mediated by inflammatory hyperactivation, and in some cases, the association may be casual rather than causal.

**Objective:** To conduct a systematic review on the cerebrovascular events in COVID-19 infection.

**Evidence review:** A comprehensive literature search on PubMed was performed including articles published from January 1, 2020, to July 23, 2020, using a suitable keyword strategy. Additional sources were added by the authors by reviewing related references. The systematic review was conducted in accordance with the PRISMA guidelines. Only articles reporting individual data on stroke mechanism and etiology, sex, age, past cardiovascular risk factors, COVID symptoms, admission NIHSS, D-dimer levels, and acute stroke treatment were selected for the review. Articles that did not report the clinical description of the cases were excluded. A descriptive statistical analysis of the data collected was performed.

**Finding:** From a total of 1,210 articles published from January 1, 2020, to July 23, 2020, 80 articles (275 patients), which satisfied the abovementioned criteria, were included in this review. A total of 226 cases of ischemic stroke (IS), 35 cases of intracranial bleeding, and 14 cases of CVST were found. Among patients with IS, the mean age was 64.16 ±14.73 years (range 27–92 years) and 53.5% were male. The mean NIHSS score reported at the onset of stroke was 15.23 ±9.72 (range 0–40). Primary endovascular thrombectomy (EVT) was performed in 24/168 patients (14.29%), intravenous thrombolysis (IVT) was performed in 17/168 patients (10.12%), and combined IVT+EVT was performed in 11/168 patients (6.55%). According to the reported presence of large vessel occlusion (LVO) (105 patients), 31 patients (29.52%) underwent primary EVT or bridging. Acute intracranial bleeding was reported in 35 patients: 24 patients (68.57%) had intracerebral hemorrhage (ICH), 4 patients (11.43%) had non-traumatic subarachnoid hemorrhage (SAH), and the remaining 7 patients (20%) had the simultaneous presence of SAH and ICH. Fourteen cases of CVST were reported in the literature (50% males), mean age 42.8 years ±15.47 (range 23–72). Treatment was reported only in nine patients; seven were treated with anticoagulant therapy; one with acetazolamide, and one underwent venous mechanical thrombectomy.

**Conclusion:** Cerebrovascular events are relatively common findings in COVID-19 infection, and they could have a multifactorial etiology. More accurate and prospective data are needed to better understand the impact of cerebrovascular events in COVID-19 infection.

## Introduction

In early December 2019, several cases of unknown origin pneumonia were described in Wuhan, the capital of Hubei Province in China (1). In less than a 4 month interval, a novel coronavirus, SARS-CoV-2 (COVID-19), was identified as the causative agent, and the infection quickly spread from China to the rest of the world, becoming a pandemic by March 2020. Since then, healthcare workers around the world have been facing a new disease with complex clinical features, far beyond the pneumonia cases that were first described in Wuhan. Indeed, the clinical syndrome of COVID-19 has shown evidence of multiorgan involvement: hematological (2), renal (3), cardiovascular (4), gastroenterological (5), dermatological (6), and neurological (7). The infection pathway of SARS-CoV-2 is mediated through angiotensin-converting enzyme 2 (ACE2), which functions as a receptor for viral infection (8). Beyond lung alveolar cells, ACE2 receptors have a wide tissue distribution in humans, including expression in the endothelium and vascular smooth muscle cells of the brain (9, 10). The full mechanism of neurologic involvement in COVID-19 remains unclear.

Reported cerebrovascular complications of COVID-19 infection—ischemic, hemorrhagic strokes, and cerebral venous sinus thrombosis (CSVT)—have been most commonly described in critically ill patients (7). It is still not clear whether cerebrovascular manifestations are caused by a direct viral action—a mechanism suggested from the retrograde brain infection from the olfactory nerve (11)—or an indirect action mediated by inflammatory hyperactivation, recognized as a cytokine storm (12), causing severe dysfunction of the immune and coagulation systems, reflected through elevated D-dimer levels and intravascular disseminated intravascular coagulation (DIC). In some patients, the presence of antibodies against cardiolipin and beta-2-glycoprotein I has been found, supporting an autoimmune mechanism (13). Another mechanism of cerebrovascular damage that has been postulated more recently, similar to what has been documented histopathologically in other organs, is through an endothelitis process (14, 15), which would account largely for the microangiopathic neuroimaging pattern described recently in a case series (16–18) and case report (19). The final cerebrovascular damage would have a neuroimaging pattern suggestive of a vasculitic process affecting the central nervous system (20, 21).

Data from postmortem brain magnetic resonance imaging (MRI) showed extensive signs of cerebrovascular involvement, including microbleeds with subcortical and posterior predominance (19). This multifocal pattern of hemorrhagic lesions could also be evocative of DIC-related lesions, leading to generalized endothelialitis (14), as also observed in ischemic stroke (IS) patients. The previously described prothrombotic scenario is at least partially correlated with the occurrence of IS and CVST. On the other hand, hemorrhagic strokes are less common but still relevant. Low platelet levels are found in patients with severe SARS-CoV-2, which may have triggered intracranial bleeding. Case reports of stroke syndromes during the COVID-19 pandemic (22–31) are heterogeneous. Most cases reveal a higher incidence of large artery atherosclerosis (LAA) stroke—indicated by greater morbidity—but also reported cases of cardioembolism (CE) and small vessel disease (SVD) (22, 28), intracranial hemorrhage, and CVST (28). The reported cases are commonly associated with comorbidities—diabetes mellitus (DM), arterial hypertension (AH), atrial fibrillation (AF), dyslipidemia (DLP), smoking, and alcohol consumption—and older patients (usually over age 60). Nonetheless, there are reports of patients of younger ages—under 40 years old—and no comorbidities (24). There are increasing data on higher levels of D-dimer and ferritin on admission—possible biomarkers of prothrombotic and inflammatory states of the disease (2, 32, 33). However, even literature data have shown that high levels of these biomarkers (mainly D-dimer) are predictors of poor prognosis and mortality (33, 34), and studies showing correlations between high levels of these biomarkers and worse stroke outcomes are still missing.

## Methods

This systematic review was conducted in accordance with the PRISMA guidelines. A comprehensive literature search on PubMed was performed including articles published from January 1, 2020, to July 23, 2020, using different combinations of the following search terms: “COVID-19,” “Coronavirus,” “Sars-Cov-2” and “neurology,” “stroke,” “ischemic stroke,” “cerebrovascular,” “intracranial hemorrhage,” “intracranial bleeding,” “subarachnoid hemorrhage,” “intracerebral hemorrhage,” and “cerebral venous sinus thrombosis.” The search was performed by two independent reviewers (FC and PF), who also performed the validity assessment. Any disagreement was resolved by consensus with a senior author (MZ). Each selected full article was further checked for cross references to additional reports. Only articles published in English were reviewed. Only articles reporting data on stroke mechanism and etiology, sex, age, past cardiovascular risk factors, COVID symptoms, admission National Institutes of Health Stroke (NIHSS) score, D-dimer levels, and acute stroke treatment were selected for the review. Articles that did not report the clinical description of the cases were excluded. Relevant qualitative and quantitative data were extracted by two authors (FC and PF) and were reviewed by a senior researcher (MZ) in the form of absolute numbers when appropriate. Where available, the data included patient demographics (age, gender), main vascular risk factors and comorbidities, COVID-19 symptoms, the time interval between COVID-19 symptom onset and stroke, NIHSS scores, the presence of large vessel occlusion (LVO), patterns of stroke on neuroimaging, relevant biological markers (D-dimer, ferritin c-reactive protein, white blood cell count, platelet count), treatment (acute recanalization and antithrombotic therapy), and outcomes. Clinical and neuroimaging data reported in text or image format were reviewed by a senior stroke neurologist (MZ), who also performed the Trial of Org 10,172 in Acute Stroke Treatment (TOAST classification) (35) whenever possible. A descriptive statistical analysis of the data collected was performed using IBM SPSS Statistics for Windows version 10.0 (IBM, Armonk, NY, USA). The results are reported as the mean ± SD.

## Results

**Figure 1:**
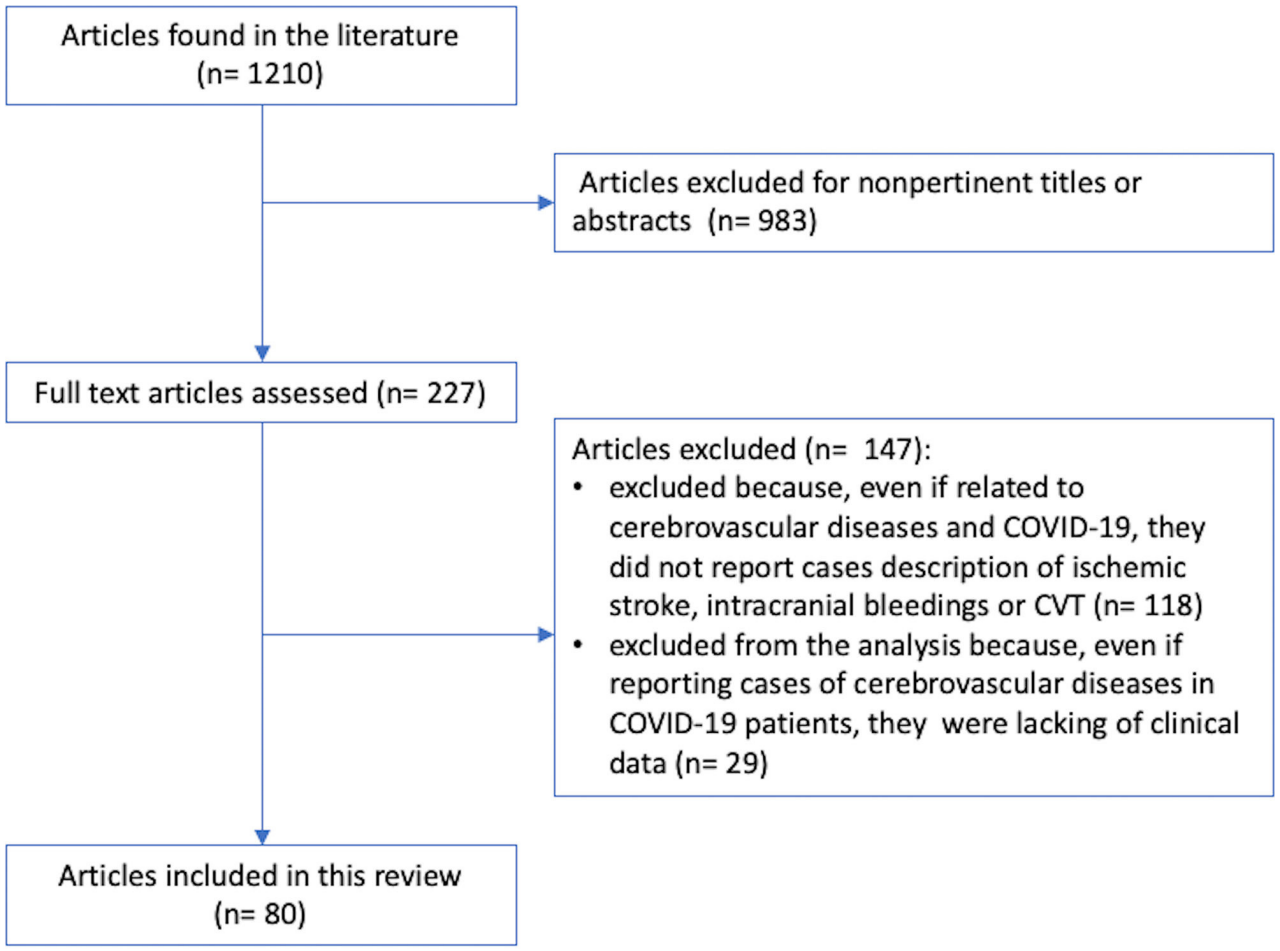
Study selection pathway.

Figure 1 shows the study selection pathway. From a total of 1,210 articles published from January 1, 2020, to July 23, 2020, 983 articles were excluded due to no pertinent titles or abstracts. Of the remaining 227 articles, 147 were excluded because even if they were related to COVID-19 and cerebrovascular diseases, they did not report case descriptions of IS, intracranial bleeding, or CVST. Of the remaining 109 articles, 29 articles (1, 7, 36–62) were excluded from the analysis because even if they reported cases of cerebrovascular diseases in COVID-19 patients, they lacked individual clinical data. The remaining 80 articles (275 patients), which satisfied the abovementioned criteria, were included in this review. We found 226 cases of patients who developed IS during COVID-19 infection (16, 18, 20, 22–29, 63–106), 35 cases of intracranial bleeding (25, 72, 74, 100–104), and 14 cases of CVST (22, 31, 105–113). Individual case descriptions are available in the Supplementary Material (S1, S2, S3).

### Ischemic Stroke

IS was reported in the literature in 226 patients, and the main features are summarized in Table 1 (full details are available in Supplementary Table 1). Among these patients, gender was reported in 188 (83.2%) patients, and 121 (53.5%) were male. Age was reported in 177 (78.3%) patients; in this subgroup, the mean age was 64.16 ± 14.73 years, with a median age of 65 years (range 27–92 years). Information about classic vascular risk factors (DM, AH, smoking, AF, alcohol consumption, chronic kidney disease [CKD], and DLP) and previous vascular history was available for 197 (87.2%) patients. Ninety-three (47.2%) patients had at least 2 vascular risk factors [age reported in 76 (38.6%) patients with mean age 69.7±12.9 years, range 39–90 years and median age 72 years]; 59 (29.9%) patients had only one vascular risk factor [age reported in 46 (23.4%) patients with mean age 60.7±12.87 years, range 36–88 years and median age 62 years]; 43 (21.8%) patients had no vascular risk factor [age reported in 34 (12.2%) patients with mean age 58.29 ± 19.01 years, range 31–92 years and median age 59 years]. Past medical history was significant for previous coronary artery disease (CAD) in 23 (10.2%) patients and for previous stroke or transient ischemic attack (TIA) in 13 (5.8%) patients. In 159 (70.35%) patients, characteristics of the COVID-19 condition were detailed (i.e., asymptomatic, mild/severe COVID infection), and among these, 24/159 (15.09%) patients were asymptomatic. No data are available about the remaining patients. For those with reported symptoms, fever, cough, and dyspnea were the most frequent. Only in 99/226 (43.81%) patients was the NIHSS score reported at the onset of stroke, and it ranged from 0 to 40 points with a mean value of 15.23 ± 9.72 points and a median of 14 points. In 164/226 (72.57%) patients, neuroimaging and their findings were reported: MRI/MRI angiography (MRA) in 28/164 (17.07%) patients and computed tomography (CT)/computed tomography angiography (CTA) in 139/164 (80%) patients. In 105/226 (46.5%) patients, large vessel occlusion (LVO) status was reported. In 23/226 (10.2%) patients, stroke etiology was not reported or not inferable from the description, according to the TOAST classification (35). In the remaining 203/226 (89.8%) patients, 131/203 (64.53%) cerebrovascular events were cryptogenic, 39/203 (19.21%) were cardioembolic, 15/203 (7.39%) were atherothrombotic, 13/203 (6.40%) were triggered by other causes (watershed stroke in systemic hypotension, posterior reversible encephalopathy syndrome, and genetic causes), and 5/203 (2.46%) were SVD-related. Among patients with reported acute treatment (168/226 patients, 74.34%), 92/168 patients (54.76%) were treated only by antithrombotic therapy (antiplatelets, low-molecular weight heparin [LMWH], oral anticoagulants). Primary endovascular thrombectomy (EVT) was performed in 24/168 patients (14.29%), intravenous thrombolysis (IVT) was performed in 17/168 patients (10.12%), and combined IVT + EVT was performed in 11/168 patients (6.55%). According to the reported presence of LVO (105 patients), 31 patients (29.52%) underwent primary EVT or bridging. Eight out of 168 (4.76%) patients underwent decompressive hemicraniectomy. Supportive treatment was performed in 24/168 patients (11.90%). The stroke treatment of 58 patients was not reported. D-dimer absolute levels were reported in 140/226 patients (61.95%) with mean value 9923.58 [±18,016] ng/mL (median 3,728; range 226–112,290). Among these patients, D-dimer levels were at least 4-fold (33, 114) higher than normal values in 99/128 patients (77.34%). D-dimer absolute levels were not reported in 86/226 patients (38.05%). Ferritin level was reported in 56/226 patients (24.78%) with mean value 1093.27 ± 1720.18 ng/mL. White blood cells (WBC) count was reported in 66/226 patients (29.2%; mean: 11,354/mm^3^; median: 8,835; SD ± 7,616; range: 100–42,900); 38/66 patients (57.6%) showed normal WBC; 25/66 patients (37.9%) showed leukocytosis and the remaining 3/66 patients showed leucopenia (4.5%). Platelet count was reported in 71/226 patients (31.4%; mean: 270,535/mm^3^; median: 239,000/mm^3^; SD ± 139,907; range: 78,000–76,2000). In particular, 45/71 patients (63.4%) showed normal platelet count; 18/71 patients (25.4%) were thrombocytopenic and the remaining 8/66 patients showed an excessive number of platelets in the blood (11.2%). C-reactive protein (CRP) levels were reported in 111/226 patients (49.1%; mean: 95.52 mg/L; median: 44.3; SD ± 100.77; range: 0.8–366.5); the majority of patients (98/111; 88.3%) presented high levels of CRP while the few remaining showed normal CRP levels (13/111; 11.7%).

**Table 1:** Characteristics of COVID-19 patients affected by ischemic stroke (IS) (*n* = 226).

**Variable**	**Valid *N***	**Values No.–%; mean [±SD]; (range)**
Age (years)	**177**	64.16 [±14.73]; (27–92)
Sex	**188**	Male 53.5%
Cardiovascular risk factors	**197**	
Arterial hypertension		102–51.78%
Diabetes mellitus		70–35.53%
Hyperlipidemia		53–26.90%
Atrial fibrillation		30–15.23%
Coronary artery disease		23–11.68%
Smoking		16–8.12%
Obesity		7–3.55%
Previous stroke or TIA	**197**	13–6.60%
NIHSS	**99**	15.23 [± 9.72]; (0–40)
COVID-19 symptoms	**159**	
Fever		81–50.94%
Cough		88–55.35%
Dyspnea		78–49.06%
Vomiting and diarrhea		3–1.89%
Body aches		9–5.66%
Asymptomatic		24–15.09%
Stroke etiology	**203**	
Cryptogenic		131–64.53%
Cardioembolic		39–19.21%
Atherothrombotic		15–7.39%
Other causes		13–6.40%
Small vessel disease		5–2.46%
Acute treatment	**168**	
Antithrombotic therapy		92–54.76%
Primary EVT		24–14.29%
Supportive treatment		20–11.90%
IVT		17–10.12%
IVT + EVT		11–14.29%
Decompressive hemicraniectomy		8–4.76%
D-Dimer (ng/mL)	**140**	9923.58 [±18,016]; (226–112,290)
Ferritin (ng/mL)	**56**	1093.27 [±1,720.18]; (7–11,062)
WBC counts (/mm3)	**66**	11,354 [±7,616]; (100–42,900)
Platelet count (/mm3)	**71**	270,535 [±139,907]; (78,000–762,000)
CRP (mg/L)	**111**	95.52 [±100.77]; (0.8–366.5)
Short-term outcome	**197**	
Survival or critically ill		129–65.48%
Death		68–34.52%

*CPR, C-reactive protein; EVT, endovascular treatment; IVT, intravenous thrombolysis; NIHSS, National Institute of Health; Stroke Scale; TIA, transient ischemic attack; WBC, white blood cells. n represents the number of cases reported in the literature*.

The available outcomes are short-term and mostly related to the hospitalization phase for acute treatment, and the outcomes of 29 patients (12.83%) are not available. A total of 129 patients (65.48%) were alive; among them, 24 patients (12.18%) were critically ill, while the remaining 68 (34.52%) were dead.

### Intracranial Bleeding

Acute intracranial bleeding was reported in 35 patients with COVID-19 in the literature (Table 2, Supplementary Table 2): 24 patients (68.57%) had intracerebral hemorrhage (ICH), 4 patients (11.43%) had non-traumatic subarachnoid hemorrhage (SAH), and the remaining 7 patients (20%) had the simultaneous presence of SAH and ICH. The mean age of the 35 patients with intracranial bleedings was 59.89 ± 11.91 years and 67.4% were males (16, 25, 28, 64, 71, 73, 107–121). Two of the 11 patients with non-traumatic SAH (regardless of the presence of ICH) were found to have a ruptured dissecting aneurysm (one in the posterior inferior cerebellar artery; one in the pericallosal artery). Concerning the ICH-only group (*n* = 24), most patients had a pre-existing risk factor for cardiovascular disease (CVD) (i.e., AH, immune thrombocytopenia, concomitant heparin treatment, hepatic cirrhosis). In contrast, five patients did not have known pre-existing risk factors for ICH. Five severely ill patients developed ICH during hospitalization in the intensive care unit as a consequence of a severe form of COVID-19. In most patients, neurological manifestations of ICH are represented by alterations in consciousness variably associated with focal signs or symptoms (i.e., motor or sensory deficits, aphasia, dysarthria).

**Table 2:** Characteristics of COVID-19 patients affected by intracranial bleeding (*n* = 35).

**Variable**	**Valid *N***	**Value No.–%; mean [±SD]; (range)**
Age (years)	**35**	59.89 [±11.91]; (30–79)
Sex	**35**	Male 67.4%
Cardiovascular risk factors	**34**	
Arterial hypertension		17–50.00%
Diabetes mellitus		7–20.59%
Coronary artery disease		3–8.82%
Hyperlipidemia		2–5.88%
Smoking		2–5.88%
Obesity		1–2.94%
Type of intracranial bleeding	**35**	
ICH		24 (68.57%)
SAH		4 (11.43%)
SAH + ICH		7 (20%)
COVID-19 symptoms	**34**	
Fever		21–61.76%
Cough		15–44.12%
Dyspnea		19–55.88%
Vomiting and diarrhea		2–5.88%
Body aches		3–8.82%
Asymptomatic		5–14.71%
ICH	**24**	68,57%
SAH	**4**	11.43%
ICH+SAH	**7**	20%
D-Dimer (ng/mL)	**18**	3,380 [±2686.82]; (410–8,961)
Ferritin (ng/mL)	**6**	2969.83 [±2861.33]; (657–8,530)
WBC counts (/mm3)	**13**	12,716 [±5,908]; (590–23,320)
Platelet count (/mm3)	**18**	217,055 [±126,445]; (1,000–510,000)
CRP (mg/L)	**17**	79.15 [±100.29]; (1–330)
Short-term outcomes	**28**	
Survival or critically ill		16–57.14%
Death		12–42.86%

*CPR, C-reactive protein; ICH, intracerebral hemorrhage; SAH, subarachnoid hemorrhage; WBC, white blood cells. n represents the number of cases reported in the literature*.

CT scan/CTA and/or Brain MRI/MRA were positive in all patients showing: supratentorial lobar ICH (13 patients); deep supratentorial ICH (four patients); cerebellar and truncal ICH (four patients), extensive supra- and infratentorial ICH (three patients). D-dimer levels were reported in 18/35 patients (51.4%; mean: 3,380 ng/ml; median: 2,876; SD ± 2686.82; range: 410–8,961), while ferritin level was reported only in 6/35 patients (17.14%) with mean value 2969.83 ± 2861.33 ng/mL. WBC was reported in 13/35 patients (37.1%; mean: 12,716/mm^3^; median: 13,600; SD ± 5,908; range: 590–23,320); 8/13 patients (61.5%) showed leukocytosis; 4/13 patients (30.8%) showed normal WBC, and the remaining patient showed leucopenia (7.7%). Platelet count was reported in 18/35 patients (51.4%; mean: 217,055/mm^3^; median: 194,000/mm^3^; SD ± 126,445; range: 1,000–510,000). In particular, 12/18 patients (66.7%) showed a normal platelet count; 5/18 patients (27.8%) were thrombocytopenic and the remaining one patient patient showed an excessive number of platelets in the blood (5.6%). CRP levels were reported in 17/35 patients (48.6%; mean: 79.15 mg/L; median: 36; SD ± 100.29; range: 1–330); the majority of patients (11/17; 64.7%) presented high levels of CRP while the few remaining showed normal CRP levels (6/17; 35.3%).

Globally, 16 patients survived while 12 died. Outcome data were not available in the remain seven patients.

### Cerebral Venous Sinus Thrombosis

Fourteen cases of CVST in patients with COVID-19 were reported in the literature (50% males, mean age: 42.8 years ± 15.47; median: 49; range: 23–72 years) (Table 3, Supplementary Table 3) (28, 39, 122–130). Seven patients did not have any known risk factors for CVST, three patients had obesity, two patients had DM, and one patient suffered from smoking and drinking consumption or a history of breast cancer in remission. Risk factors were not reported in two patients. The main symptoms associated with COVID-19 were fever (8 patients), cough (7 patients), dyspnea (4 patients), vomiting and diarrhea (1 patient), and body aches (1 patient). Data were not reported in three patients, while one patient did not develop any symptoms. The NIHSS was available only in two patients; in the majority of patients, neurological manifestations of CVST were headache and/or altered mental status variably associated with focal signs or symptoms (i.e., motor or sensory deficits, aphasia, altered vision) and seizures (one patient). CT scan/CTA and/or brain MRI/MRA were positive in 13 patients (in one patient, neuroimaging data were not available). In particular, venous infarction with hemorrhagic transformation was detected in 9/14 patients (64.29%), while in three patients, no parenchyma alterations were found. The transverse sinus was involved in six patients, the straight sinus in four patients, the sigmoid sinus and the vein of Galen in three patients, and the superior sagittal sinus in two patients. D-dimer levels were available in 8/14 (57.14%) and were elevated in each of them (mean value: 4624.5 ± 5783.16 ng/mL; median: 2,618 ng/mL; range: 902–18,431 ng/mL). Ferritin level was reported in 2/14 patients (14.28%) with mean value 1233.5 ± 238 ng/mL. WBC count was reported in 8/14 patients (57.14%) with mean value 12,337 ± 5,233/mm3 and platelet count was available in 6/14 patients (42.86%) with mean value of 179,500 ± 109,381/mm3. Mean CPR levels were 95.93 ± 75.40 mg/L in 4/14 patients (28.57%). Treatment was reported only in nine patients; seven of them were treated with anticoagulant therapy (LMWH, unfractionated heparin [UFH], or direct oral anticoagulants). One patient was treated only with acetazolamide, and one patient underwent venous mechanical thrombectomy. Five patients (50%) died due to complications of CVST and COVID-19 infection. Outcome data were not available for three patients.

**Table 3:** Characteristics of COVID-19 patients affected by cerebral venous sinus thrombosis (CVST) (*n* = 14).

**Variable**	**Valid *N***	**Value No.–%; mean [±SD]; (range)**
Age (years)	**14**	42.8 [±15.47]; (23–72)
Sex	**14**	Male 50.0%
Cardiovascular risk factor	**12**	
None		7–58.33%
Obesity		3–24.00%
Diabetes mellitus		2–15.67%
Arterial hypertension		1–8.33%
Smoking		1–8.33%
COVID-19 symptoms	**11**	
Fever		8–72.73%
Cough		7–63.64%
Dyspnea		4–36.36%
Vomiting and diarrhea		1–9.09%
Body aches		1–9.09%
Asymptomatic		1–9.09%
Sinus and vein involvement	**13**	
Transverse sinus		6–46.15%
Straight sinus		4–30.77%
Sigmoid sinus		3–23.08%
Vein of Galen		3–23.08%
Superior sagittal sinus		2–15.38%
Hemorrhagic transformation	**9**	64.29%
D-dimer (ng/mL)	**8**	4624.5 [±5,783]; (902–18,431)
Ferritin (ng/mL)	**2**	1233.5 [±238]; (1,040–1,427)
WBC counts (/mm3)	**8**	12,337 [±5,233]; (6,300–20,220)
Platelet count (/mm3)	**6**	179,500 [±109,381]; (42,000–335,000)
CRP (mg/L)	**4**	95.93 [±75.40]; (20–170.8)
Short-term outcomes	**11**	
Death		6–54.55%
Survival or critically ill		5–45,45%

*CPR, C-reactive protein; WBC, white blood cells. n represents the number of cases reported in the literature*.

## Discussion

It is well known and characterized in the literature that both acute and chronic infections and inflammatory states can be triggers of stroke (131, 132). In particular, it has been proposed that respiratory tract infection may act as a trigger and increase the risk of large vessels and/or cardioembolic IS, especially in subjects without vascular risk factors (133). In particular, influenza-like illness has previously been associated with an increased stroke risk (134). The risk of stroke during the COVID-19 outbreak was compared to the risk of stroke during the influenza outbreak in the previous year in a cohort study (135), showing that 1.6% of emergency department (ED)-admitted COVID-19 patients had acute IS vs. 0.2% of patients with influenza, reflecting an odds ratio of 7.6 (95% CI, 2.3–25.2).

Therefore, cerebrovascular events are relatively common findings in COVID-19 infection, and they could have a multifactorial etiology. The causal association with COVID-19 infection is not clearly evident or inferable in all the cases described. The manifestations are multifaceted, and the neuroimaging pattern of the patients is also consistent with different pathophysiological mechanisms, so it is difficult to identify a single pattern of cerebrovascular disease related to COVID-19.

More information is available on thrombotic events than on intracranial bleeding. IS and CVST could have a common pathophysiological path in the inflammatory and pro-coagulant state correlated with COVID-19 and is supported from the biochemical point of view by the significantly increased values of D-dimer. In some cases of CVST, however, COVID-19 infection is explicitly reported as a possible etiological cofactor in association with known risk conditions, such as taking estrogen-progestin therapy (73, 128). Moreover, the presence of known previous risk factors for stroke (e.g., AH, AF, vascular disease, DLP, smoking) is common in reported cases of IS, but there are also reports among patients with no known risk factors (26.4%, Supplementary Table 1).

### Ischemic Stroke

The age of IS patients on whom data are available is substantially in line with that of patients with a higher incidence of cerebrovascular events (136), also in association with multiple vascular risk factors present in the described cohort. In fact, the average age of patients with IS without any pre-existing vascular risk factor was 62.9 ± 17.2 years, with a median age of 67.5 years.

A temporal association between COVID-19 and cerebrovascular events is presented in all reported case reports; sometimes, in particular in cases of stroke caused by LVO without atherothrombosis, an etiopathogenetic association has been hypothesized between COVID-19-related coagulopathy and stroke, as in the case of CVST. Not all patients were able to collect information on the presence of an LVO, and similarly, the NIHSS score was not reported except in a limited number of patients.

Although an NIHSS score threshold has never been demonstrated that is capable of differentiation with sufficient accuracy for emergency treatment, it is nevertheless sufficiently agreed that a score >10 is associated with a greater probability of finding an LVO. Furthermore, the main limitations of this approach derive from the fact that low NIHSS scores cannot exclude LVO, not that high scores are not predictive of LVO (137). In reported patients, the NIHSS score is at least 10, and in the whole sample, a high rate of documented LVO, often in multiple vessels, was reported.

Additionally, in many of the patients for whom it was not possible to have information on the state of patency of the large cerebral vessels, ischemic lesions in multiple arterial territories have been reported, and in some of them, the etiological definition according to the TOAST classification was determined by the evidence of cardiac embolic sources (AF, endocarditis, dilated heart disease), which does not exclude the possibility that COVID-19 may have acted as a trigger on known vascular risk factors. Similar reasoning is possible for cases in which the etiological category “atherothrombosis of large vessels” is defined by the presence of a thrombotic burden, often very extensive and superimposed on an atheroma, as well as the fact that the documentation of this pattern of vascular imaging is relatively rare in acute IS treatment cases in comprehensive stroke centers (138).

Cryptogenic stroke was the most common subtype of IS in COVID-19 patients, and it is an interesting fact that can be interpreted in the context of the inflammatory and prothrombotic state characteristic of the disease, with documentation of arterial and venous thrombosis, micro- and macrovascular thrombosis, and other body areas (139–141). In fact, in many patients with COVID-19, the final cause of death has been documented to be a thrombotic complication, particularly a pulmonary embolism. Moreover, in some cases reported as cardioembolic, mostly due to the already known history of AF or hypokinetic heart disease with severe left ventricular function deficit, patients with LVO stroke have been described despite ongoing anticoagulation therapy. It is therefore possible that in this case, the prothrombotic component linked to the infection may have played a role, at least in association with the known risk factors. The cases described with ischemia in multiple vascular territories, even in the absence of LVO, could also fall within the context of cryptogenic cerebral embolism. Considering the high percentage of patients described with cryptogenic LVO, it can be speculated that the prothrombotic mechanism linked to COVID-19 can act both in isolation and in association with the classic vascular risk factors, regardless of age.

Regarding individual ischemic lesions with SVD patterns, the role of causes other than COVID-19 appears more probable, which may have played a triggering role, as is known for many systemic or localized infectious events (142).

The high percentage of patients with high D-dimer values, often >4 times the normal values, may indirectly corroborate the hypothesis of the role of the prothrombotic mechanism linked to COVID-19 in a significant proportion of patients with IS reported thus far with sufficient detail in the literature.

Tang and colleagues (34) reported that in a series of hospitalized patients with COVID-19 pneumonia 71.4% of non-survivors and 0.6% survivors met the criteria of DIC during their hospital staying and had coagulation abnormalities with markedly elevated D-dimer levels. We can therefore speculate that the occurrence of both thrombosis in cerebral large vessels, often multiple, and thrombotic microangiopathy, as neuroimaging data suggest, is one of the main mechanisms by which COVID-19 has an etiological association with stroke. It should also be considered that, even in the absence of COVID-19, among the medical emergencies associated with markedly high levels of D-dimer in ED, cerebrovascular events are second only to sepsis for D-dimer level and the D-dimer correlates with mortality (143). It is therefore possible that the diagnostic and prognostic role of D-dimer values as a coagulopathy marker in these patients should be specified by the dosage of other biomarkers (for example endothelial damage) and there is no clear information on the prognostic role of the variation of D-dimer levels over time (144).

The therapeutic approach to this mechanism is therefore mostly similar to that of stroke with LVO in revascularization strategies but more empirical in the subsequent acute and post-acute phases with a variable combination of antithrombotic drugs, often LMWH, with variable dosage.

The etiopathogenetic link appears less immediate for SVD-related cerebrovascular events. In some reported cases in patients with severe COVID-19 infection and evidence of neurological involvement after several unsuccessful attempts of extubation (27), brain MRI provided evidence of an unusual pattern of microbleeds, predominantly affecting the corpus callosum, and punctiform lesions that were DWI-positive in the centrum semiovale. Both thrombotic microangiopathy related to direct or indirect damage by SARS-CoV-2 on the endothelium of cerebral small vessels and brain-blood-barrier injury related to hypoxemia have been hypothesized. An emblematic case of the possible causality of the association between COVID-19 and IS in a patient with CADASIL is reported (95). The few described cases of IS caused by spontaneous carotid dissection also fall into the TOAST etiopathogenetic category of “other determined etiology,” which collects data on the known rare causes of IS. In this case, it is possible to postulate the role of COVID-19 infection as a dissection trigger, similar to what is known for respiratory tract infections in general (145, 146), with the possibility of two further specific elements of COVID-19 infection or the prothrombotic potential and tropism for the vascular endothelium (147). A consideration that deserves attention is that IS has been reported in patients with significant differences in the severity of COVID-19, and in some cases (90), it represented the reason for access to the hospital; that is, it is not a limited event that is more severe with severe respiratory failure and requires ventilatory support. A further element that conditions the association between COVID-19 and co-occurrent IS is that the severity of the respiratory picture and of the infection in general is widely different in various stages of the course of the disease, which can influence the prognosis of cerebrovascular events, both in the acute phase and in the post-acute phase, and the global outcome.

In a multicenter case study related to all consecutive patients hospitalized with laboratory-confirmed COVID-19 and IS in 28 sites from 16 countries (148), which collected 174 patients, it is suggested that these patients have a worse functional outcome and higher mortality than stroke patients without COVID-19 hospitalized in the same period. This worse prognosis can be correlated with the increased stroke severity at admission in COVID-19 associated stroke patients compared with the non-COVID-19 cohort and with the broad multi-system complications of COVID-19.

All these factors make it very complex to define the best IS therapy for these patients, both in the acute phase (IVT, EVT, or both) and in secondary prevention, with the need to treat the vascular complications of other organs with anti-thrombotic therapy.

Also, in the treatments described (Table 1, Supplementary Table 1), the outcome in terms of the recanalization of the previously occluded vessel is reported only occasionally, even more rarely the final outcome in terms of final brain parenchymal damage and functional outcome (e.g., modified Rankin scale). It is therefore not possible at the moment to make considerations that go beyond the individual case on this point.

### Intracranial Hemorrhage

In general, there are insufficient data to be able to make etiopathogenetic hypotheses on intracranial bleeding, given the small number of cases described, the presence of an increased risk of bleeding related to the need for antithrombotic therapy (mainly anticoagulant treatment), and the different characterization of SAH and ICH. As already described for some subtypes of IS, it is possible to postulate that COVID-19 infection may in some cases have acted as a contributing cause or trigger, for example, in patients with SAH due to dissecting aneurysms, as known for endocarditis (149), but in general, the infectious hypothesis of aneurysm rupture was rejected several years ago (150). The systemic characteristics of the disease and the DIC type of multiorgan involvement pattern could, in some of the reported cases, have a close causal relationship with ICH.

### Cerebral Venous Sinus Thrombosis

In CVST patients, the infectious trigger and hypercoagulability are well-known causal links. Hypercoagulability is a known complication of COVID-19 (125). Indeed, it has already been reported that COVID-19 infection may predispose patients to thrombotic disease, both in the venous and arterial circulations, due to excessive inflammation, platelet activation, endothelial dysfunction, and stasis (151). Moreover, there is also a growing understanding that antiphospholipid antibodies (anticardiolipin IgA and anti-β2-glycoprotein I IgA and IgG) may play a role in both arterial and venous infarcts in COVID-19 patients (123). Most likely, through a multifactorial process, the virus could lead to a hypercoagulable state that is responsible, at least in part, for both respiratory and cerebral involvement (152).

### Limitations

This analysis has limitations, deriving primarily from the possibility that cerebrovascular events in patients with COVID-19 are underreported, especially in patients in critical clinical conditions but also in asymptomatic or paucisymptomatic patients presenting mild stroke-related symptoms and not evaluated by a neurologist. Another consideration is that many patients are expected to have remained undiagnosed because they did not have access to hospital facilities during the period of greatest pandemic burden (153).

Furthermore, the data extracted from the cases reported in the literature are often incomplete and very heterogeneous, which further limits the strength of the results of the analysis. In particular, detailed information on the diagnostic path and the treatment carried out, as well as on the evolution of the cerebrovascular event and COVID-19, are not often available. Even the patient's outcome is not always reported. Even with these limitations, the analysis of the available data shows an image of the daily clinical reality experienced in hospitals during the pandemic. The description of the cases and the reported conclusions are affected by the clear limitation of the quality of the data reported in the literature on the etiological work-up of patients with cerebrovascular events in the context of a global health crisis such as the pandemic in progress. For this reason, the data must be interpreted with caution, and what is described must be confirmed by prospective studies with greater completeness of the collected data, e.g., the European Academy of Neurology planned registry, Ean NEuro-covid ReGistrY (ENERGY) (154).

Other considerations concern the revascularization treatment of patients with IS and COVID-19. Although COVID-19 itself is not a contraindication for thrombolysis or endovascular treatment, usually worse clinical status, the unavailability of resources, and a delayed time window of intervention make these treatments impossible. It has been recently reported in the French national registry of IVT and EVT for stroke that only 10/1,513 treated patients had biologically proven COVID-19 infection at 7/32 centers (33, 37). In the same registry (41, 45) a significant decrease in patients treated with EVT during the first stages of the COVID pandemic was reported with alarming indicators of lengthened care delays. Similar results, although with a progressive improvement in the treatment's time metrics, have been reported in the New York City series (60).

### Perspectives for Stroke Management

Some lessons may be derived from the review of the case series of patients with cerebrovascular and COVID-19 events available in the literature. SARS-CoV-2 has been revealed as the “great imitator” due its variety of clinical presentations. Hence, any acute neurologic symptoms, especially cerebrovascular-related symptoms, must be considered a “potential” COVID-19 syndrome manifestation.

Firstly, we need to consider that the disease burden of cerebrovascular diseases remains even in the COVID-19 pandemic and should be addressed in a timely manner, preserving the stroke code from the extensive changes in disease management pathways seen in several countries (136, 155). Measures of social distancing or lockdown are not reasons to avoid or delay the assessment of suspected stroke patients in emergency departments. There are fewer reports from centers where there was a reduction of patients with stroke diagnosis due possible “fear of becoming infected” in the hospital and an increase in cases outside of the time window for reperfusion therapy (31, 45). These situations increase poor outcomes, disabilities, and long-term impacts on healthcare and social security.

The way stroke care has been affected during the pandemic has made it necessary to highlight the special measures of the “Protected Code Stroke” guidelines (156). These measures include crisis management resources, screening recommendations, and personal protective equipment (PPE). The COVID-19 pandemic addresses a need to go beyond normal code stroke triage, which includes information to help define reperfusion strategies (e.g., time of onset of the symptoms, presence or absence of absolute contraindications), but it now also includes new features: infection control (symptoms and clinical signs initially, as well as minimal laboratory screening) and contact with patients confirmed to have or suspected of having COVID-19.

Concurrent with required neurovascular imaging, chest CT scans add important information to infection control—findings suggestive of COVID-19 are present up to 82% of patients (1)—implicate low risk to patients and teams and add only a small amount of time to examination (e.g., minutes).

The use of PPE during the stroke code is mandatory to teams and patients. If not intubated, a surgical mask must be placed on the patient during transport and evaluations. Concerns regarding team PPE are related to the risk of aerosolization during the procedures. If not present, droplet and contact PPE are sufficient: full-sleeved gown, surgical mask, eye protection, and gloves. However, if there is a risk of aerosolization, equipment must be added to prevent airborne transmission, such as the use of N95 or PFF2 masks and face shields. It is fundamental to correctly evaluate the situation and use the proper PPE, thus avoiding the unnecessary and wasted use of equipment in the context of scarce resources or a lack of protection, if needed, revealing a false sense of security of the team.

Decisions concerning reperfusion therapy were previously discussed, but cleaning protocols must be followed in imaging and angiographic suite rooms. Thus, decontamination of the patient area and the surrounding zone and equipment between procedures may be required by specialized teams to prevent transmission to the next patient and possibly imply a delay of care for other patients (157, 158), especially in low-resource settings where usually only one piece of equipment is available. Another proposed hypothesis is to use tenecteplase instead of alteplase for IVT in some cases to reduce EC spread of COVID-19 (159).

After the decision of reperfusion therapy or conservative treatment, it is reasonable to consider the high risk of contamination of healthcare workers in stroke care units or intensive care units to reduce the exposure of the team to this avoidable risk. Then, we suggest reducing the number of health professionals in close contact with the patient and to eventually increase the intervals between the clinical revaluations after reperfusion therapy, as recommended in standard protocols.

During hospitalization, rehabilitation planning is a key part of after-stroke care. Physical therapy, occupational therapy, and speech therapy should not be withheld, but therapy services must be wisely considered when appropriate and not selected indiscriminately—alternative strategies focusing on self-exercises could increase their effectiveness and empower the patient toward their treatment. Telehealth could also be used by pharmacists, stroke education nurses, and dietary consultants and prevent unnecessary direct contact with patients (160).

Assessing stroke etiology is central to determining the best approach in the secondary prevention of new events. Due to the prothrombotic state, despite low evidence, especially with thrombotic events or high D-dimer levels, screening for lupus anticoagulant and antiphospholipid antibodies could be routinely inserted and could add information for a definition of full-intensity anticoagulation (13, 161).

However, it should be kept in mind that in patients treated in the ED due to the occurrence of stroke symptoms (and not for COVID-19 symptoms), the diagnosis of SARS-CoV-2 infection could be delayed or missed (this was particularly true during the first phases of the outbreak). Indeed, the literature cited in this review are mainly related to previously diagnosed COVID-19 patients who developed acute cerebrovascular diseases and stroke patients in whom the diagnosis of COVID-19 has been made directly in the ED. In contrast, the monitoring of stroke patients in which the diagnosis of SARS-CoV-2 infection has been carried out during the course of hospitalization may be difficult, which also leads to interpretation difficulties (i.e., hospital transmission). These challenges could be responsible for the underestimation of COVID-related strokes. Considering the abovementioned limitations, we decided to focus this review on strokes in COVID-19 patients and not to address the more complex and broad issue of the relationship between stroke and COVID-19 infection.

## Conclusions

Cerebrovascular events are relatively common findings in COVID-19 infection, and they could have a multifactorial etiology. In patients directed to the ED due to the appearance of stroke symptoms (and not for COVID-19 symptoms), the diagnosis of SARS-CoV-2 infection could be delayed or missed (this was particularly true during the first phases of the outbreak). Considering the abovementioned limitations, more accurate and prospective data (such as those currently collected from many ongoing international registries) are needed to better understand the impact of cerebrovascular events in COVID-19 infection.

## Data Availability Statement

The original contributions presented in the study are included in the article/Supplementary Material, further inquiries can be directed to the corresponding author.

## Disclosure

FC received personal fees from Zambon outside the submitted work. EM has received honoraria from Abbott, Medtronic, and Newronika for consulting and lecturing; she has received an educational grant from Boston Scientific. All other authors declare no financial disclosures.

## Author Contributions

PF, CG, and MZ were responsible for writing the manuscript. EM, FC, and MZ were responsible for its drafting. CG, EM, FC, and MZ was responsible for its revision. All authors read and approved the final manuscript.

## Conflict of Interest

The authors declare that the research was conducted in the absence of any commercial or financial relationships that could be construed as a potential conflict of interest.
