# Supplementary material for: COVID-19 and Cerebrovascular Diseases: A Systematic Review and Perspectives for Stroke Management
Source: Front Neurol. 2020 Nov 5;11:574694. doi: 10.3389/fneur.2020.574694 (PMC7674955; doi:10.3389/fneur.2020.574694)
Supplement: Supplementary file 1 [file Table_1.docx]

| **Author** | **Sex** | **Age** | **CVD Risk Factors** | **Previous stroke/TIA** | **Previous CAD** | **COVID symptoms** | **NIHSS admission** | **Neurological manifestation** | **BRIN MRI/CT abnormalities** | **LVO** | **Mechanism of stroke** | **Treatment of Stroke** | **D-Dimer (ng/mL)** | **Ferritin (ng/mL)** | **WBC counts (/mm3)** | **Platelet count (/mm3)** | **CRP (mg/L)** | **Outcomes** |
| --- | --- | --- | --- | --- | --- | --- | --- | --- | --- | --- | --- | --- | --- | --- | --- | --- | --- | --- |
| Beyrouti et al^22^ | M | 64 | None | None | None | Cough, dyspnea, fever, myalgia, loss of appetite | NA | Word-finding difficulties, bilateral incoordination, right homonymous hemianopia | Brain MRI: acute left VA thrombus, acute left PICA territory infarction with petechial haemorrhagic transformation. 7 days later, DWI MRI showed bilateral acute PCA territory infarcts | Yes | Undertermined | LMWH | > 80000 | 4927 | 6750 | 305000 | 305,4 | Survival |
| Beyrouti et al^22^ | F | 53 | AH, DM, AF, CHF | None | None | Malaise, cough, dyspnea, fever | NA | Acute confusion, incoordination, RC | Brain CT: acute right parietal cortical and left cerebellar infarct with mass effect and hydrocephalus | NA | Cardioembolic | LMWH | 7750 | 1853 | 23050 | 254000 | 150,1 | Death |
| Beyrouti et al^22^ | M | 85 | AH, AF, DLP, CAD | None | 1 | Cough | NA | Dysarthria, right facial droop, right-sided weakness | Brain CT: hyperdensity consistent with thrombus in the left PCA and acute infarction in the left temporal stem and cerebral peduncle | Yes | Cardioembolic | Apixaban | 16100 | 1027 | 5080 | 173000 | 161,2 | Survival |
| Beyrouti et al^22^ | M | 61 | AH, stroke, CVI | 1 | 1 | Fever, cough, dyspnea, tachypnoea | NA | Dysarthria, left facial droop, left-sided weakness | Brain MRI: acute infarction in the R corpus striatum suggesting transient occlusion of the right M1 MCA; FLAIR MRI: established infarct in the same region with moderate background cerebral small vessel disease | No | Undertermined | LMWH | 27190 | 1167 | 8970 | 408000 | 12,8 | Survival |
| Beyrouti et al^22^ | M | 83 | AH, DM, CAD, Smoking, Alcohol | None | 1 | Fever, cough, dyspnea,fatigue | NA | Dysarthria, left facial droop, left-sided weakness and left-sided sensory inattention | CT and CTA: thrombotic occlusion of a proximal M2 branch of the R MCA. Repeat CT at 24 hours: focus of parenchymal low density involving the R insular cortex in keeping with an evolving right MCA territory infarct | Yes | Undertermined | IVT | 19450 | NA | 11030 | 197000 | 27,7 | Survival |
| Beyrouti et al^22^ | M | 73 | None | None | None | Dyspnea e tachypnoea | NA | Aphasia, right facial droop and right-sided weakness | Brain MRI: acute infarction in the right thalamus, left portion of the pons, right occipital lobe and right cerebellar hemisphere. Time-of-flight images showed thrombotic material in the basilar artery and bilateral mild- to-moderate P2 PCA stenosis | Yes | Undertermined | IVT | 1080 | 655 | 7300 | 403000 | 179,9 | Survival |
| Gonzalez Pinto et al^23^ | F | 36 | Smoking | None | None | NA | 21 | Global aphasia, right hemiplegia | CTA: occlusion of the left ICA, MCA and left ACA with a free- floating thrombus in the ascending aorta with no signs of aortic atheromatosis | Yes | Undertermined | None | 7540 | NA | 23600 | NA | 156 | Death |
| Oxley et al^24^ | F | 33 | None | None | None | Cough, headche, chills | 19 | left Hemiplegia, left facial droop, gaze preference, homonymous hemianopia, dysarthria, sensory deficit | Brain CT, CTA: partial infarction of the right MCA with a partially occlusive thrombus in the right ICA at the cervical bifurcation | Yes | Undertermined | Apixaban | 460 | 7 | 7800 | 427000 | NA | Survival |
| Oxley et al^24^ | M | 37 | None | None | None | None | 13 | RC, dysphasia, right hemiplegia, dysarthria, sensory deficit | NA | NA | Undertermined | EVT + Apixaban | 5200 | 136 | 9900 | 299000 | NA | Survival |
| Oxley et al^24^ | M | 39 | AH, DLP | None | None | none | 16 | RC, gaze preference to the right, left homonymous hemianopia, left hemiplegia, ataxia | NA | NA | Undertermined | EVT + Aspirin | 2300 | 1564 | 5500 | 135000 | NA | Survival |
| Oxley et al^24^ | M | 44 | DM | None | None | Lethargy | 23 | RC, global dysphasia, right hemiplegia, gaze preference | NA | NA | Undertermined | IVT + EVT + hemicraniectomy + aspirin | 13800 | 987 | 9000 | 372000 | NA | Survival |
| Oxley et al^24^ | M | 49 | Stroke, DM | 1 | None | Fever, cough, lethargy | 13 | RC, left hemiplegia, dysarthria, facial weakness | NA | NA | Undertermined | EVT + stent + aspirin + clopidrogrel | 1750 | 596 | 4900 | 255000 | NA | Survival |
| Al Saiegh et al^25^ | F | 62 | NA | NA | NA | NA | NA | right hemiparesis and aphasia | CTA: left MCA occlusion | Yes | Undertermined | EVT | NA | NA | NA | NA | NA | Survival |
| Moshayedi et al^26^ | M | > 80 | CAD | NA | 1 | Dyspnea | NA | Aphasia, gaze preference to left, right hemiplegia, right hemianopia | CTA: occlusion of the left M1 MCA | Yes | Undertermined | Supportive treatment | NA | NA | NA | NA | NA | Survival |
| Lodigiani et al^29^ | M | 67 | Methastic lung cancer | None | None | NA | NA | NA | NA | Yes | Undertermined | EVT | NA | NA | NA | NA | NA | Survival |
| Lodigiani et al^29^ | M | 67 | None | None | None | NA | NA | NA | NA | NA | - | Aspirin | NA | NA | NA | NA | NA | Survival |
| Lodigiani et al^29^ | F | 76 | None | None | None | NA | NA | NA | NA | NA | - | Aspirin + Nadroparin | NA | NA | NA | NA | NA | Death |
| Lodigiani et al^29^ | F | 64 | None | None | None | NA | NA | NA | NA | NA | - | IVT + aspirin + Nadroparin | NA | NA | NA | NA | NA | Survival |
| Lodigiani et al^29^ | M | 68 | None | None | None | NA | NA | NA | NA | NA | - | Clopidogrel + Nadroparin | NA | NA | NA | NA | NA | Survival |
| Lodigiani et al^29^ | M | 69 | AF | None | None | NA | NA | NA | NA | Yes | Cardioembolic | EVT + Aspirin + Anticoagulant | NA | NA | NA | NA | NA | Survival |
| Lodigiani et al^29^ | M | 57 | None | None | None | NA | NA | NA | NA | NA | - | Heparin | NA | NA | NA | NA | NA | Survival |
| Lodigiani et al^29^ | M | 73 | AF | None | None | NA | NA | NA | NA | NA | Cardioembolic | IVT + Nadroparin | NA | NA | NA | NA | NA | Survival |
| Lodigiani et al^29^ | F | 75 | None | None | None | NA | NA | NA | NA | NA | - | Nadroparin | NA | NA | NA | NA | NA | Death |
| Yaghi et al.^63^ | NA | NA | None | None | None | Cough | NA | NA | NA | Yes | Undertermined | IVT + EVT + Aspirin | >10000 | NA | NA | NA | 11 | Survival |
| Yaghi et al.^63^ | NA | NA | AH, DM, DLP, AF, CHF, CAD | None | 1 | Cough, fever, hypoxia | NA | NA | NA | Yes | Large artery atherosclerosis (basilar stenosis) | Aspirin, clopidogrel | 342 | NA | NA | NA | 31,6 | Death |
| Yaghi et al.^63^ | NA | NA | AH, DM, AF, DLP, prior stroke | 1 | None | Fever, cough, hypoxia | NA | NA | NA | No | Cardioembolic | IVT | 3247 | NA | NA | NA | 72 | Death |
| Yaghi et al.^63^ | NA | NA | AH, DLP | None | None | Cough, hypoxia | NA | NA | NA | NA | Undertermined | Anticoagulation | >10000 | NA | NA | NA | 111 | Death |
| Yaghi et al. ^63^ | NA | NA | AH | None | None | Fever, cough, hypoxia | NA | NA | NA | NA | Undertermined | Anticoagulation | >10000 | NA | NA | NA | 63,8 | Death |
| Yaghi et al. ^63^ | NA | NA | AH, DM, DLP | None | None | none | NA | NA | NA | Yes | Large artery atherosclerosis (M1 segment stenosis) | Aspirin NA Clopidogrel | NA | NA | NA | NA |  | Survival |
| Yaghi et al. ^63^ | NA | NA | AH, DM, DLP | None | None | Hypoxia | NA | NA | NA | Yes | Cardioembolic | EVT NA Aspirin | 769 | NA | NA | NA | 16,7 | Survival |
| Yaghi et al. ^63^ | NA | NA | NA | NA | NA | Fever, cough, hypoxia | NA | NA | NA | NA | Undertermined | Anticoagulation | >10000 | NA | NA | NA | 170 | Death |
| Yaghi et al. ^63^ | NA | NA | AH, AF | None | None | none | NA | NA | NA | NA | Cardioembolic | Anticoagulation | NA | NA | NA | NA | 6,9 | Survival |
| Yaghi et al. ^63^ | NA | NA | AH, DM, DLP | None | None | Fever, cough, hypoxia | NA | NA | NA | NA | Undertermined | Anticoagulation | 319 | NA | NA | NA | 101,1 | Death |
| Yaghi et al. ^63^ | NA | NA | DLP | None | None | Fever, cough, hypoxia | NA | NA | NA | NA | Undertermined | Anticoagulation | 4841 | NA | NA | NA | 214,3 | Critically Ill |
| Yaghi et al. ^63^ | NA | NA | AH, AF | None | None | Cough, hypoxia | NA | NA | NA | Yes | Cardioembolic | IVT + EVT Anticoagulation | >10000 | NA | NA | NA | 94,4 | Critically Ill |
| Yaghi et al. ^63^ | NA | NA | AH, DLP | None | None | Fever, cough, hypoxia | NA | NA | NA | Yes | Undertermined | IVT + EVT Anticoagulation | 4102 | NA | NA | NA | 154,4 | Death |
| Yaghi et al. ^63^ | NA | NA | None | None | None | Cough | NA | NA | NA | NA | Undertermined | Anticoagulation | >10000 | NA | NA | NA | 141,8 | Death |
| Yaghi et al. ^63^ | NA | NA | DM, DLP | None | None | Hypoxia | NA | NA | NA | NA | Undertermined | Anticoagulation | >10000 | NA | NA | NA | 38,8 | Survival |
| Yaghi et al. ^63^ | NA | NA | AH, AF, DLP | None | None | Fever, cough, hypoxia | NA | NA | NA | NA | Cardioembolic | Anticoagulation | 3723 | NA | NA | NA | 76 | Death |
| Yaghi et al. ^63^ | NA | NA | AH, AF, DLP, CAD | None | 1 | Fever, cough, hypoxia | NA | NA | NA | NA | Cardioembolic | Aspirin | 2004 | NA | NA | NA | 17 | Death |
| Yaghi et al. ^63^ | NA | NA | AH, DM, AF, DLP, CAD | None | 1 | Fever, cough, hypoxia | NA | NA | NA | NA | Cardioembolic | Anticoagulation | 226 | NA | NA | NA | 108 | Survival |
| Yaghi et al. ^63^ | NA | NA | AH, DLP | None | None | Fever, cough | NA | NA | NA | NA | Undertermined | Aspirin | 662 | NA | NA | NA | 60,3 | Survival |
| Yaghi et al. ^63^ | NA | NA | DM, DLP | None | None | Fever, cough, hypoxia | NA | NA | NA | NA | Undertermined | Anticoagulation | 4786 | NA | NA | NA | 70 | Death |
| Yaghi et al. ^63^ | NA | NA | AH, DLP, CAD | None | 1 | Fever, cough, hypoxia | NA | NA | NA | NA | Undertermined | Anticoagulation | 5870 | NA | NA | NA | 248 | Death |
| Yaghi et al. ^63^ | NA | NA | None | None | None | Fever, cough, hypoxia | NA | NA | NA | NA | Undertermined | Anticoagulation | 3081 | NA | NA | NA | 323,73 | Death |
| Yaghi et al. ^63^ | NA | NA | AH | None | None | none | NA | NA | NA | Yes | Undertermined | EVT NA Anticoagulation | 2735 | NA | NA | NA | 11,9 | Critically Ill |
| Yaghi et al. ^63^ | NA | NA | DM | None | None | Fever, cough, hypoxia | NA | NA | NA | NA | Undertermined | Anticoagulation | 2814 | NA | NA | NA | 142,1 | Death |
| Yaghi et al. ^63^ | NA | NA | None | None | None | Fever, cough, hypoxia | NA | NA | NA | NA | Undertermined | Anticoagulation | 3248 | NA | NA | NA | 9,9 | Critically Ill |
| Yaghi et al. ^63^ | NA | NA | DLP | None | None | Fever, cough, hypoxia | NA | NA | NA | NA | Undertermined | Anticoagulation | >10000 | NA | NA | NA | 297 | Critically Ill |
| Yaghi et al. ^63^ | NA | NA | None | None | None | Fever, cough, hypoxia | NA | NA | NA | NA | Undertermined | Anticoagulation | 2703 | NA | NA | NA | 314,5 | Critically Ill |
| Yaghi et al. ^63^ | NA | NA | DM, CAD | None | None | Fever, cough, hypoxia | NA | NA | NA | NA | Watershed from hypotension | Anticoagulation | >10000 | NA | NA | NA | 366,5 | Critically Ill |
| Yaghi et al. ^63^ | NA | NA | AH, DLP, CHF | None | None | Fever, cough, hypoxia | NA | NA | NA | NA | Watershed from hypotension | Anticoagulation | 2087 | NA | NA | NA | 235 | Critically Ill |
| Yaghi et al. ^63^ | NA | NA | None | None | None | Fever, cough, hypoxia | NA | NA | NA | NA | Undertermined | Anticoagulation | 6933 | NA | NA | NA | 210,78 | Survival |
| Yaghi et al. ^63^ | NA | NA | None | None | None | Fever, cough, hypoxia | NA | NA | NA | NA | Undertermined | Anticoagulation | >10000 | NA | NA | NA | 284,91 | Critically Ill |
| Yaghi et al. ^63^ | NA | NA | DLP | None | None | Fever, cough, hypoxia | NA | NA | NA | NA | Undertermined | Anticoagulation | 3058 | NA | NA | NA | 83,03 | Critically Ill |
| Morassi et al. ^64^ | M | 64 | Smoking, CAD | None | 1 | Fever, cough, hypoxia | NA | neurological deterioration, with inability to arouse during sedation lightening. | Brain CT: multiple hypodense lesions involving different cortical and subcortical regions of both cerebral hemispheres | NA | Undertermined | NA | 7744 | NA | NA | 78000 | 175 | Death |
| Morassi et al. ^64^ | M | 75 | AH, DM | None | None | cough, fever | NA | At the beginning left hemiparesis. On day 21, the patient’s neurological status further declined, with a Glasgow Coma Scale (GCS) of 3. | Brain CT and CTA: cortico-subcortical hypodensity over the right cingulate gyrus with occlusion of the right pericallosal artery. New brain CT: multiple, bilateral, supratentorial (right frontal and parietal, left perirolandic and bilateral occipital) and infra-tentorial (vermian, left cerebellar hemisphere) ischemic lesions | Yes | Undertermined | NA | NA | NA | NA | NA | 46 | Death |
| Morassi et al. ^64^ | M | 82 | AH, DM, previous TIA | 1 | None | cough, fever, dyspnea | NA | left hemiparesis | Brain CT: new small hypodense area in the right thalamus of presumed ischemic origin. CTA: no intra-luminal filling defect in the carotid arteries, vertebrobasilar system, and other intra-cranial vessels | No | Small-vessel | aspirin and clopidogrel | NA | NA | NA | NA | 181 | Death |
| Morassi et al. ^64^ | F | 76 | AH, DM, aortic valve replacement, stroke | 1 | None | cough, fever | NA | episodes of transient loss of consciousness, followed by confusion | Brain MRI: small rounded area of diffusion restriction on the left pre- rolandic gyrus. Brain MRI angiography: no alterations of intracranial vessels. | No | Undertermined | NA | 1381 | NA | NA | NA | 12 | Critically Ill |
| Fara et al. ^65^ | F | 33 | None | None | None | none | NA | numbness of her left hand which quickly progressed to left hemiplegia and hemisensory loss. | CTA: non-occlusive thrombus in the right CCA. Brain MRI: acute stroke within the territory of the right MCA. | Yes | Undertermined | Apixaban | normal | NA | NA | NA | NA | NA |
| Fara et al. ^65^ | M | 77 | AH, DLP, and bilateral thromboses of the deep leg veins discovered two months prior, prescribed aspirin, clopidogrel, and warfarin (although subtherapeutic on presentation) | None | None | cough | NA | aphasia, left hemiparesis | CTA: thrombus within the distal right CCA | Yes | Undertermined | Enoxaparin 1 mg/kg every 12 hours | significantly elevated DNAdimer levels | NA | NA | NA | NA | NA |
| Fara et al. ^65^ | M | 55 | DM | None | None | none | NA | Isolated left hand weakness which progressed to weakness of the left face, arm, and leg. | CTA: thrombosis within the right CCA extending into the ICA, which appeared occlusive or near-occlusive | Yes | Undertermined | Acuterly eptifibatide intraarterially followed by subsequently heparin infusion | normal | NA | NA | NA | NA | NA |
| Valderrama et al. ^66^ | M | 52 | AH | None | None | shortness of breath, cough, fever. | 20 | global aphasia, left gaze preference, and right partial hemianopia, facial weakness, severe hemiparesis, and hemianesthesia | CTA: left intracranial ICA occlusion. Brain CT: early infarct signs of in the left basal ganglia, internal capsule, caudate head, insular ribbon, operculum, and right posterior frontal lobe. CTP: favorable mismatch ratio of 4.1. | Yes | Undertermined | aspirin | >10000 | NA | NA | NA | 11 | Survival |
| Zayet et al. ^67^ | M | 84 | DM, AH, CAD, PAD, AF | None | 1 | dyspnea, cough | NA | dysarthria, left hemiplegia, RC | Brain MRI: acute ischemic stroke in multiple vascular areas | No | Cardioembolic | apixaban switched to IV unfractionated heparin [18 UI/kg/h]). | NA | NA | NA | NA | NA | Death |
| Zayet et al. ^67^ | M | 74 | history of multiple cardiovascular diseases, AF | None | None | influenzaNAlike illness, confusion | NA | nonfluent aphasia | Brain CT: many recent ischemic infarctions in different vascular areas; brain MRI confirmed this finding | No | Cardioembolic | IV unfractionated heparin (18 UI/kg/h) | NA | NA | NA | NA | NA | Survival |
| Wang et al. ^68^ | NA | NA | CAD | NA | 1 | NA | 27 | NA | CTA: left inferior MCA branch occlusion and tandem thrombus in the left carotid bulb | Yes | Undertermined | EVT | elevated DNAdimer | NA | NA | NA | elevated CRP | Death |
| Wang et al. ^68^ | NA | NA | None | None | None | NA | 29 | NA | CTA: left ICA terminus occlusion in conjunction with a left pulmonary artery embolus | Yes | Undertermined | EVT | elevated DNAdimer | NA | NA | NA | elevated CRP | Death |
| Wang et al. ^68^ | NA | NA | CAD | NA | 1 | NA | 28 | NA | CTA: partial basilar artery occlusion. Cerebral angiography: BA thrombosis and concomitant left cervical ICA occlusion | Yes | Undertermined | EVT | elevated DNAdimer | NA | NA | NA | elevated CRP | Death |
| Wang et al^68^ | NA | NA | AH, obesity, DM | None | None | NA | 10 | NA | CTA: right ICA terminus occlusion and thrombus in the right carotid bulb | Yes | Undertermined | EVT | elevated DNAdimer | NA | NA | NA | elevated CRP | Survival |
| Wang et al. ^68^ | NA | NA | None | None | None | NA | 20 | NA | CTA: left M1 MCA occlusion | Yes | Undertermined | EVT | elevated DNAdimer | NA | NA | NA | elevated CRP | Critically Ill |
| Lima et al. ^69^ | F | 89 | None | None | None | fluNAlike symptoms | NA | right hemiparesis, aphasia, dysarthria | CTA: left M2 MCA occlusion | Yes | Undertermined | NA | NA | NA | NA | NA | NA | NA |
| Goldberg et al. ^70^ | M | 64 | AH | None | None | Fever, myalgia, shorteness of breath | NA | left hemiparesis | CTA: markedly decreased vascular flow within the branches of the right MCA and bilateral ACAs | No | Undertermined | NA | Elevated DNAdimer | Elevated Ferritin | Elevated WBC count | NA | NA | Death |
| Reddy et al. ^71^ | F | 59 | AH, DM | None | None | NA | NA | Paralyzed | CT: subacute left frontal and occiptal infarcts | NA | Undertermined | Supportive treatment | 11300 | 1188 | 17400 | 220000 | NA | Survival |
| Reddy et al. ^71^ | M | 47 | AH, DM, DLP | None | None | NA | 18 | left hemiplegia | Brain CT: right parietal, temporal and occipital infarctions | NA | Undertermined | Supportive treatment | 4430 | NA | 6900 | 183000 | NA | Survival |
| Reddy et al. ^71^ | F | 39 | AH | None | None | NA | 10 | right hemiparesis | CTA: left MCA occlusion | Yes | Undertermined | IVT + EVT | 2380 | 84 | 9890 | 465000 | NA | Survival |
| Reddy et al. ^71^ | M | 78 | AH, DM, AF | None | None | NA | 10 | NA | NA | NA | Cardioembolic | Supportive treatment | 3010 | NA | 11600 | 131000 | NA | Critically Ill |
| Reddy et al. ^71^ | M | 54 | AH, DM, DLP | None | None | NA | 14 | NA | NA | NA | - | Supportive treatment | 10160 | 1633 | 7000 | 137000 | NA | Critically Ill |
| Reddy et al. ^71^ | F | 39 | None | None | None | NA | NA | Paralyzed | NA | NA | Carotid dissection | NA | 1100 | 32,5 | 6560 | 256000 | NA | Survival |
| Reddy et al. ^71^ | M | 76 | AH, DM, Cancer | None | None | NA | 2 | NA | NA | NA | Undertermined | NA | 1530 | 639 | 8190 | 214000 | NA | Survival |
| Reddy et al. ^71^ | F | 65 | AH, DM, DLP | None | None | NA | 11 | NA | NA | NA | Undertermined | NA | 5500 | 11062 | 6480 | 249000 | NA | Survival |
| Reddy et al. ^71^ | M | 62 | DM | None | None | NA | NA | Paralyzed | NA | NA | Undertermined | Supportive treatment | 5120 | 600,5 | 2540 | 121000 | NA | Survival |
| Sharifi-Razavi et al^72^ | F | 88 | AH | None | None | Fever | NA | right peripheral facial paresis, left limb paresis, nystagmus | Brain CT: low-density lesion at right cerebellar hemipshere | No | Undertermined | NA | NA | NA | 17700 | NA | 63 | Death |
| Sharifi-Razavi et al^72^ | F | 85 | None | None | None | Asthenia, cough | 22 | left hemiplegia and central facial paresis | Brain CT: attenuation and effacement at the right hemisphere around the Sylvian fissure | NA | Undertermined | NA | NA | NA | 5100 | NA | 29 | NA |
| Sharifi-Razavi et al^72^ | M | 55 | CAD, AH | None | 1 | Cough, fever | NA | Broca’s aphasia, right central facial palsy, and right hemiplegia | Brain CT: hypodensity at left basal ganglia | NA | Undertermined | NA | NA | NA | 19500 | NA | 30 | NA |
| Franceschi et al. ^73^ | F | 59 | AH, obesity | None | None | Cough and dyspnea | NA | right hemineglect | Brain CT: focal low attenuation in the left posterior frontal/anterior parietal lobes consistent with an infarction | NA | Undertermined | NA | elevated DNAdimer | elevated Ferritin | NA | NA | elevated CRP | Death |
| Franceschi et al. ^73^ | M | 33 | None | None | None | NA | NA | left hemiparesis and numbness, right force-gazed deviation | Brain CT: subacute infarctions in the right frontal, parietal, and parieto-occipital regions with edema and mass effect resulting in subfalcine herniation and an associated small hemorrhage in the R parietal region | No | Undertermined | IVT | > 50000 | 1636 | NA | NA | 312 | Critically Ill |
| Franceschi et al. ^73^ | M | 71 | DM, DLP, AH | None | None | Fever, cough | NA | NA | Brain CT: large left MCA infarction with evolving low attenuation in the left frontal, parietal, and temporal lobes extending to the basal ganglia | Yes | Undertermined | NA | 31000 | NA | NA | NA | NA | Death |
| Franceschi et al. ^73^ | M | 40 | AH, DM, DLP | None | None | Cough | NA | RC, bilateral loss of vision, headache | Brain CT: low attenuation in the parietal and temporal lobes compatible with acute infarcts. CTA: occlusion of the right ICA extending to the supraclinoid segment. CTA of the neck demonstrated filling defects in the distal common carotid arteries extending to the bulbs and external carotid arteries. There was occlusion of the right ICA at the C2 level. | Yes | Undertermined | Anticoagulation | 3115 | NA | NA | 762000 | NA | Critically Ill |
| Franceschi et al. ^73^ | F | 37 | obesity | None | None | Respiratory failure | NA | right upper extremity weakness, slurred speech, headaches | Brain CT, CTA: intraluminal thrombus extending from the left CCA into the proximal ICA | Yes | Undertermined | Anticoagulation | NA | NA | NA | NA | NA | Survival |
| Franceschi et al. ^73^ | M | 71 | AH, AF | None | None | Fever, cough, dyspnea | NA | NA | Brain MRI: acute and subacute infarcts in the frontal lobes (right side greater than left side), bilateral centrum semiovale, right corona radiata, occipital lobes, and left cerebellar hemisphere | No | Cardioembolic | NA | NA | 602 | NA | NA | 25 | Critically Ill |
| Franceschi et al. ^73^ | F | 62 | AH obesity, DM, DLP | None | None | Cough, fever | NA | RC | Brain CT: low attenuation in the left occipital lobe | Yes | Undertermined | Anticoagulation | 12000 | Elevated Ferritin | NA | NA | elevated CRP | Survival |
| Franceschi et al. ^73^ | M | 73 | None | None | None | Cough, fever | NA | RC, seizures | Brain CT: focal low attenuation in the occipital and right frontal lobes, compatible with infarctions | NA | - | NA | 27000 | NA | NA | NA | 25 | Death |
| Franceschi et al. ^73^ | F | 62 | Cancer | None | None | Respiratory failure | NA | acute encephalopathy | Brain CT: low attenuation in the right frontal, temporal, and parietal regions consistent with infarction | Yes | Undertermined | NA | 5628 | Elevated Ferritin | 12000 | NA | elevated CRP | Death |
| Gill et al. ^74^ | F | 79 | AH, DLP, DM | None | None | Tachypnea, hypoxemia | 21 | Aphasia, left hemiparesis | CTA (CTA) of the head and neck demonstrated a partial right M2-M3 MCA occlusion | Yes | Undertermined | EVT | 8240 | NA | NA | NA | 50 | Survival |
| Viguier et al. ^75^ | M | 73 | None | None | None | Fever, cough | 10 | Aphasia, R hemiparesis | brain CT, CTA: subtle cortical left frontal hypoattenuation with more extended surrounding hypoperfusion and distal occlusion of branch. Large intraluminal floating thrombus appended to a hypoattenuated non-stenosing plaque of the left CCA wall | Yes | Large artery | Anticoagulation | 2220 | 1096 | 500 | normal | 219 | Survival |
| Sangalli et al. ^76^ | F | 75 | AF, CKD, AH, DLP, Obesity | None | None | Fever, cough, dyspnea | 8 | Global aphasia, right hemiparesis | Brain CT: left frontotemporal and parietal ischaemic lesion | No | Cardioembolic | IVT | NA | NA | 6800 | 182000 | 19,4 | Death |
| Sangalli et al. ^76^ | M | 53 | AH, DLP | None | None | Cough, Myalgia | 5 | left sensorimotor hemisyndrome | - | No | Undertermined | IVT | 313 | 89 | 7300 | 251000 | 2,9 | Survival |
| Sangalli et al. ^76^ | M | 77 | DM | None | None | Dyspnea | 10 | Global aphasia, mild right hemiparesis | Brain CT with CTP: complete CBV/MTT mismatch without early ischaemic changes | No | LAA | IVT | NA | NA | 7500 | 132000 | 4,4 | Death |
| Sangalli et al. ^76^ | M | 59 | Smoking | None | None | Cough, dyspnea | 9 | Global aphasia | Brain CT: bilateral both ischaemic and haemorrhagic lesions | No | Cardioembolic (infective endocarditis on Valve prosthesis) | IVT | 112290 | NA | 21500 | 197000 | 221 | Death |
| Sparr et al. ^77^ | F | 84 | AH, DM | None | None | respiratory failure, acute renal failure requiring hemodialysis | NA | RC and failure to wean from the ventilator after 4 days off sedation | Brain CT: hypodensity of the splenium of the corpus callosum | NA | Undertermined | NA | 6510 | NA | 8400 | NA | 19,7 | Critically Ill |
| Sparr et al. ^77^ | F | 52 | AH, DM | None | None | respiratory failure, acute renal failure requiring hemodialysis | NA | RC and transient downward eye deviation on day 16 on a ventilator | Brain CT: hypodense lesion of the splenium of the corpus callosum | NA | Undertermined | NA | 11400 | NA | 24400 | NA | 3,6 | Survival |
| Sparr et al. ^77^ | F | 62 | DM | None | None | persistent cough followed by hypoxia | NA | acute confusion | Brain MRI: multiple infarctions involving the cerebral and cerebellar hemispheres bilaterally and the right side of the splenium of the corpus callosum | NA | Undertermined | apixaban | 20000 | NA | 9800 | NA | 11,1 | Survival |
| Sparr et al. ^77^ | F | 54 | DM | None | None | respiratory distress with prolonged intubation and renal failure requiring dialysis | NA | RC | Brain MRI: edema of the hemispheres bilaterally with cortical petechiae, most pronounced in the parietal and temporal lobes, also affecting frontal lobes and splenium of the corpus callosum. | NA | Undertermined | NA | 5150 | NA | 18300 | NA | 22,5 | Death |
| Kremer et al. ^18^ | M | 86 | AH, DLP | None | None | NA | NA | left hemiplegia/RC | Brain CT: large artery infarction | Yes | - | NA | NA | NA | NA | NA | NA | NA |
| Kremer et al. ^18^ | M | 71 | AH, DM | None | None | NA | NA | left hemiplegia | Brain CT: large artery infarction | Yes | Cardioembolic | NA | 2860 | NA | NA | NA | NA | NA |
| Kremer et al. ^18^ | F | 74 | NA | None | None | NA | NA | Bilateral pyramidal tract signs | Brain CT: watershed cerebral infarction | No | Rare causes | NA | 20000 | NA | NA | NA | NA | NA |
| Kremer et al. ^18^ | M | 63 | smoking | None | None | NA | NA | left-sided weakness /left-sided sensory inattention | Brain CT: large artery infarction | No | Cardioembolic | NA | 1000 | NA | NA | NA | NA | NA |
| Kremer et al. ^18^ | M | 65 | AH, DM, DLP, stroke | 1 | None | NA | NA | RC | Brain CT: watershed cerebral infarction | No | Rare causes | NA | 1570 | NA | NA | NA | NA | NA |
| Kremer et al. ^18^ | F | 78 | AH | None | None | NA | NA | Dysarthria | Brain CT: watershed cerebral infarction | No | Rare causes | NA | 720 | NA | NA | NA | NA | NA |
| Kremer et al. ^18^ | F | 75 | AH, DLP, AF | None | None | NA | NA | Aphasia, right hemiplegia, RC, Agitation | Brain CT: large artery infarction | Yes | Undertermined | NA | NA | NA | NA | NA | NA | NA |
| Kremer et al. ^18^ | M | 79 | AH, DLP, stroke | 1 | None | NA | NA | Headaches, RC, Confusion | Brain CT: watershed cerebral infarction | No | Rare causes | NA | NA | NA | NA | NA | NA | NA |
| Kremer et al. ^18^ | F | 71 | AH, smoking, AF | None | None | NA | NA | left hemiplegia | Brain CT: large artery infarction | Yes | Cardioembolic | NA | 20000 | NA | NA | NA | NA | NA |
| Kremer et al. ^18^ | M | 59 | AH, smoking | None | None | NA | NA | left hemiplegia, left facial droop | Brain CT: large artery infarction | Yes | Undertermined | NA | 2868 | NA | NA | NA | NA | NA |
| Kremer et al. ^18^ | M | 66 | AH, DLP, DM | None | None | NA | NA | left pyramidal tract signs, left facial droop, Aphasia | Brain CT: large artery infarction | Yes | Dissection | NA | 5227 | NA | NA | NA | NA | NA |
| Kremer et al. ^18^ | M | 72 | DLP, AF | None | None | NA | NA | RC | Brain CT: large artery infarction | No | Cardioembolic | NA | 993 | NA | NA | NA | NA | NA |
| Kremer et al. ^18^ | M | 78 | AH | None | None | NA | NA | Dysarthria, right-sided weakness, right facial droop | Brain CT: large artery infarction | No | Undertermined | NA | NA | NA | NA | NA | NA | NA |
| Kremer et al. ^18^ | M | 76 | NA | None | None | NA | NA | Confusion | Brain CT: watershed cerebral infarction | No | Rare causes | NA | 3977 | NA | NA | NA | NA | NA |
| Kremer et al. ^18^ | F | 92 | NA | None | None | NA | NA | Confusion | Brain CT: watershed cerebral infarction | No | Rare causes | NA | 987 | NA | NA | NA | NA | NA |
| Kremer et al. ^18^ | F | 77 | AH, stroke | 1 | None | NA | NA | left hemiplegia, left homonymous hemianopia, Headaches | Brain CT: large artery infarction | Yes | Cardioembolic | NA | NA | NA | NA | NA | NA | NA |
| Kremer et al. ^18^ | M | 88 | AH, DM, DLP, AF, stroke | 1 | None | NA | NA | right hemiplegia, RC | Brain CT: large artery infarction | Yes | Cardioembolic | NA | NA | NA | NA | NA | NA | NA |
| Diaz-Segarra et al. ^78^ | M | 54 | AH | None | None | Cough, fever, chills | NA | Dysarthria, left hemiparesis, RC | Brain MRI: sustained basilar and right SCA infarctions | Yes | Undertermined | EVT, aspirin | 7835 | 451 | 18200 | 244000 | 28 | Death |
| Diaz-Segarra et al. ^78^ | M | 37 | DM | None | None | none | NA | Aphasia, facial droop; right hemiparesis, sensory deficit, complete hemianopsia | Brain MRI: left MCA infarction | Yes | Large artery | IVT, mechanical EVT, aspirin | 3918 | 703 | 8000 | 489000 | 114 | Survival |
| Diaz-Segarra et al. ^78^ | M | 65 | DM | None | None | Shortness of breath, fever | NA | RC; Unable to assess deficits further | Brain MRI: numerous scattered punctate foci of restricted diffusion primarily within the subcortical white matter of the bilateral cerebral hemispheres | No | Undertermined | Aspirin | 6384 | 884 | 22400 | 170000 | 310 | Death |
| Diaz-Segarra et al. ^78^ | F | 68 | AH | None | None | Cough, shortness of breath, diarrhea | NA | RC; Unable to assess deficits further | Brain CT: right PCA ischemia | No | Undertermined | Aspirin | 2484 | 1062 | 42900 | 506000 | 252 | Survival |
| Hernández-Fernández et al.^16^ | M | 58 | AH, obesity | None | None | fever | 28 | NA | CTA: distal BA occlusion | Yes | Undertermined | IVT | NA | NA | NA | 260000 | 10 | Survival |
| Hernández-Fernández et al. ^16^ | M | 83 | AH, DLP, CAD, rheumatic valve disease, AF | None | 1 | none | 22 | NA | Brain CT: Bilteral cerebellar, left thalamus - occipital infarction. Angio-CT: left P1 PCA occlusion | Yes | Cardioembolic | Supportive treatment | 990 | NA | NA | 160000 | 146 | Death |
| Hernández-Fernández et al. ^16^ | M | 56 | Smoking | None | None | none | 20 | NA | Brain CT, CTA: Left MCA extensive infarction, left M1 MCA occlusion | Yes | Undertermined | IVT + EVT | 4273 | 268 | NA | 181000 | 2 | Survival |
| Hernández-Fernández et al. ^16^ | M | 65 | Smoking, AF | None | None | none | 16 | NA | Brain CT, CTA: Right frontal-temporal infarction with right M1 MCA occlusion | Yes | Cardioembolic | EVT | 721 | 289 | NA | 151000 | 15,3 | Death |
| Hernández-Fernández et al. ^16^ | M | 75 | None | None | None | none | 16 | NA | Brain CT, CTA: Right parietal and thalamus, left frontal infarctions | No | Undertermined | Supportive treatment | 669 | 110 | NA | 174000 | NA | Survival |
| Hernández-Fernández et al. ^16^ | M | 65 | None | None | None | none | NA | RC | Vertebro-basilar complete infarction. BA occlusion | Yes | Undertermined | EVT | 2316 | 788 | NA | 399000 | 96 | Death |
| Hernández-Fernández et al. ^16^ | M | 56 | AH | None | None | none | 5 | NA | Brain MRI: left MCA watershed infarction, left parietal ischemia. Transcranial ultrasound: focal distal left MCA stenosis | Yes | Atherosclerotic | Supportive treatment | 457 | 884 | NA | 330000 | 29,3 | Survival |
| Hernández-Fernández et al. ^16^ | M | 76 | DM, smoking, AH, DLP, COPD, B12 vitamin deficit | None | None | fever, dyspnea | 26 | NA | Brain CT: right insular subacute infarction. Right MCA hyperdensity and proximal M1 MCA occlusion | Yes | Undertermined | Supportive treatment | 828 | 645 | NA | 636000 | 39 | Death |
| Hernández-Fernández et al. ^16^ | F | 79 | AH, DM | None | None | none | 18 | NA | Brain CT: right MCA infarction; right M1 MCA occlusion | Yes | Undertermined | IVT + EVT (TICI 3) | 90404 | 258 | NA | 191000 | 60 | Survival |
| Hernández-Fernández et al. ^16^ | M | 62 | AH | None | None | none | 26 | NA | Brain CT: Cerebellar Infarction, CTA: basilar occlusion and Left V1 VA dissection | Yes | Other determined etiology | EVT (TICI 2C) | 918 | 267 | NA | 167000 | 7 | Survival |
| Hernández-Fernández et al. ^16^ | F | 86 | AH, DM | None | None | none | 9 | NA | Brain CT: left insular infarction | No | Undertermined | Supportive treatment | 21863 | 155 | NA | 239000 | 49 | Death |
| Hernández-Fernández et al. ^16^ | M | 65 | DM, DLP | None | None | Dyspnea | 6 | NA | right parietal slow perfusion | No | Undertermined | Supportive treatment | 1028 | 1697 | NA | 216000 | 11 | Survival |
| Hernández-Fernández et al. ^16^ | F | 51 | DM, DLP | None | None | Fever | 4 | seizures | right PCA slow perfusion. Brain MRI: no ischemic lesions | No | Undertermined | IVT | 962 | 49 | NA | 253000 | 0,8 | Survival |
| Hernández-Fernández et al. ^16^ | M | 90 | AH, AF, CAD, intermittent claudication | None | 1 | Fever | 14 | NA | Brain CT: subcortical malacia, leukoaraiosis, hyperdensity MCA. | Yes | Cardioembolic | Supportive treatment | 3518 | 1334 | NA | 303000 | 27 | Death |
| Hernández-Fernández et al. ^16^ | M | 54 | AH | None | None | Cold | 0 | NA | Brain CT: chronic bilateral subcortical hypodensities. right hemispheric hypoperfusion. Brain MRI: diffuse multiple microhemorrages, bilateral ischemic lesions. Angiography: signs of vasculitis alla vascular territories. Control CT: mild bilateral SAH | No | Other determined etiology | EVT | 369 | 433 | NA | 239000 | 14 | Survival |
| Hernández-Fernández et al. ^16^ | M | 48 | None | None | None | none | 8 | NA | Brain CT: right parietal subacute infarction. M2 MCA occlusion. Right bulbar carotid dissection | Yes | Other determined etiology | Supportive treatment | 361 | 329 | NA | 294000 | 3,6 | Survival |
| Hernández-Fernández et al. ^16^ | F | 85 | AH, DM, COPD, CAD, AF | None | 1 | none | 22 | NA | Brain CT: complete infarction MCA , hyperdensity MCA, proximal M1 MCA occlusion | Yes | Cardioembolic | Supportive treatment | NA | 282 | NA | 280000 | 39 | Death |
| Chen et al. ^79^ | F | 72 | AH | None | None | fever | NA | left hemiparesis, dysarthria | Brain CT: negative | No | Cardioembolic | Supportive treatment | NA | NA | NA | NA | NA | Survival |
| Liang et al. ^80^ | M | 57 | AH, DM, AF, CHF | None | None | Hypoxia | 18 | NA | CTA: left M1 MCA occlusion | Yes | Cardioembolic | Decompressive Hemicraniectomy | NA | NA | NA | NA | NA | Death |
| Liang et al. ^80^ | F | 51 | AH, DM | None | None | Fever, cough | 20 | NA | CTA: left ICA occlusion | Yes | Undertermined | Decompressive Hemicraniectomy | NA | NA | NA | NA | NA | Death |
| Liang et al. ^80^ | M | 61 | DM | None | None | hypoxia | 17 | NA | CTA: left ICA occlusion | Yes | Undertermined | IVT + EVT (TICI 2B) + decompressive Hemicraniectomy | NA | NA | NA | NA | NA | Death |
| Liang et al. ^80^ | M | 61 | DM | None | None | none | 12 | NA | CTA: right ICA occlusion | Yes | Undetermined | IVT + EVT (TICI 2B) + decompressive Hemicraniectomy | NA | NA | NA | NA | NA | Survival |
| Liang et al. ^80^ | M | 41 | DM | None | None | hypoxia | 23 | NA | CTA: left M1 MCA occlusion | Yes | Undetermined | IVT + EVT (TICI 2B) + decompressive Hemicraniectomy | NA | NA | NA | NA | NA | Survival |
| Liang et al. ^80^ | M | 57 | None | None | None | Fever, cough, hypoxia | 22 | NA | CTA: left M1 MCA occlusion | Yes | Undetermined | Decompressive Hemicraniectomy | NA | NA | NA | NA | NA | Survival |
| Liang et al. ^80^ | M | 53 | CAD, ckd | None | 1 | hypoxia | 12 | NA | CTA: right M1 MCA occlusion | Yes | Undetermined | Decompressive Hemicraniectomy | NA | NA | NA | NA | NA | Death |
| Fitsiori et al. ^81^ | M | 65 | AH, DM, DLP, COPD, SAS | None | None | Shortness of breath | NA | Confusion | Brain MRI: two acute lacunar infarcts in subcortical wmhite Matter | No | small-vessel | NA | NA | NA | NA | NA | NA | NA |
| Paterson et al. ^82^ | M | 61 | NA | NA | NA | NA | NA | NA | MRI: acute infarct in the right striatum. Multiple supra- and infratentorial cortical and subcortical microhaemorrhages | No | - | LMWH | 27190 | NA | NA | NA | NA | Survival |
| Paterson et al. ^82^ | M | 64 | NA | NA | NA | NA | NA | NA | MRI: (1st event): acute left VA thrombus and acute left PICA territory infarction with microhaemorrhages. 2nd event - 7 days later: bilateral acute PCA territory infarcts despite therapeutic anticoagulation | Yes | Undetermined | LMWH | 80000 | NA | NA | NA | NA | Survival |
| Paterson et al. ^82^ | M | 64 | NA | NA | NA | NA | NA | NA | MRI: subacute infarcts within the deep internal border zones of the cerebral hemispheres bilaterally, and within the left frontal white matter. Background moderate small vessel disease and established cortical infarcts, in arterial border zone territories | No | Undetermined | LMWH | 29000 | NA | NA | NA | NA | Critically Ill |
| Paterson et al. ^82^ | F | 53 | NA | NA | NA | NA | NA | NA | Brain CT: acute right parietal cortical and left cerebellar infarct with mass effect and hydrocephalus | No | Undetermined | LMWH | 7750 | NA | NA | NA | NA | Death |
| Paterson et al. ^82^ | M | 58 | NA | NA | NA | NA | NA | NA | MRI: extensive evolving left MCA infarction with evidence of petechial haemorrhage and associated mass-effect. Persistent occlusion of the left M2 MCA branches | Yes | Undetermined | LMWH | 75320 | NA | NA | NA | NA | Survival |
| Paterson et al. ^82^ | M | 85 | NA | NA | NA | NA | NA | NA | Brain CT: hyperdensity consistent with thrombus in the left PCA and acute infarction in the left temporal lobe, brainstem and cerebral peduncle | Yes | Undetermined | aspirin 7 days then switched to apixaban | 16100 | NA | NA | NA | NA | Survival |
| Paterson et al. ^82^ | M | 73 | NA | NA | NA | NA | NA | NA | MRI: acute infarction in the right thalamus, left pons, right occipital lobe and right cerebellar hemispheres | No | Undetermined | Aspirin 5 days then switched to LMWH | NA | NA | NA | NA | NA | Survival |
| Paterson et al. ^82^ | F | 27 | NA | NA | NA | NA | NA | NA | CT: right MCA and right ACA territory infarction | Yes | Undetermined | Aspirin 10 days then LMWH | NA | NA | NA | NA | NA | Survival |
| Saggese et al. ^83^ | M | 62 | AH, DM, CAD | None | 1 | fever, cough | 18 | right hemiplegia, expressive aphasia with partial sparing of comprehension, right neglect | Brain CT and CTA: no parenchymal lesions (ASPECT score 10) and no vascular intracranial or extracranial occlusions. A control Brain CT, 24 h after onset: left frontotemporal hypodense area consistent with acute ischemia | No | Undetermined | IVT | 1510 | NA | 14500 | 263000 | 53,8 | Survival |
| Mahboob et al. ^84^ | F | 58 | stroke | 1 | None | none | 15 | left homonymous hemianopia, right gaze preference, left-sided facial droop, left-sided hemiplegia | CTA: negative. Brain MRI: left cerebellar infarction in the PICA territory of the PICA. | No | Undetermined | IVT | NA | NA | 100 | NA | NA | Death |
| Mohamud et al. ^85^ | M | 55 | NA | NA | NA | NA | 24 | NA | left ICA bifurcation + left ICA petrous segment occlusion | Yes | Undetermined | EVT (TICI 2A) | NA | NA | NA | NA | NA | Survival |
| Mohamud et al. ^85^ | F | 78 | NA | NA | NA | NA | 25 | NA | left ICA bifurcation occlusion | Yes | NA | NA | NA | NA | NA | NA | NA | Survival |
| Mohamud et al. ^85^ | M | 62 | NA | NA | NA | NA | 25 | NA | right ICA bifurcation + right M1 MCA occlusion | Yes | NA | NA | NA | NA | NA | NA | NA | Survival |
| Mohamud et al. ^85^ | M | 74 | NA | NA | NA | NA | 5 | NA | right ICA bifurcation occlusion | Yes | NA | NA | NA | NA | NA | NA | NA | Survival |
| Mohamud et al. ^85^ | M | 59 | NA | NA | NA | NA | 1 | NA | right ICA bifurcation + right M2 MCA occlusion | Yes | NA | NA | NA | NA | NA | NA | NA | Survival |
| Mohamud et al. ^85^ | M | 67 | NA | NA | NA | NA | 30 | NA | left ICA bifurcation + left M1 MCA occlusion | Yes | NA | EVT (TICI 2B) | NA | NA | NA | NA | NA | Survival |
| Gulko et al. ^86^ | F | 53 | NA | NA | NA | NA | NA | confusion | Brain MRI: acute-subacute infarcts in the right frontal and parietal lobes. CTA: intraluminal thrombus in the proximal right ICA | Yes | NA | NA | NA | NA | NA | NA | NA | NA |
| Gulko et al. ^86^ | NA | NA | NA | NA | NA | NA | NA | acute aphasia, right-sided weakness | CTA: left MCA M1 occlusion, intraluminal thrombus in the proximal left ICA and carotid bifurcation | Yes | NA | NA | NA | NA | NA | NA | NA | NA |
| Li et al. ^28^ | F | NA | NA | NA | NA | severe | 3 | - | - | Yes | atherosclerotic | Antiplatelet | NA | NA | NA | NA | NA | Survival |
| Li et al. ^28^ | M | NA | NA | NA | NA | severe | 3 | - | - | No | small-vessel | Antiplatelet | NA | NA | NA | NA | NA | Survival |
| Li et al. ^28^ | M | NA | NA | NA | NA | Non severe | 4 | - | - | Yes | atherosclerotic | Antiplatelet | NA | NA | NA | NA | NA | Survival |
| Li et al. ^28^ | M | NA | smoking | None | None | severe | 8 | - | - | NA | Cardioembolic | Antiplatelet | NA | NA | NA | NA | NA | Death |
| Li et al. ^28^ | F | NA | NA | NA | NA | severe | 20 | - | - | Yes | Atherosclerotic | Antiplatelet | NA | NA | NA | NA | NA | Death |
| Li et al. ^28^ | M | NA | smoking | None | None | severe | 2 | - | - | No | small-vessel | Antiplatelet | NA | NA | NA | NA | NA | Death |
| Li et al. ^28^ | F | NA | NA | NA | NA | Non severe | 23 | - | - | NA | Cardioembolic | Anticoagulant | NA | NA | NA | NA | NA | Death |
| Li et al. ^28^ | F | NA | NA | NA | NA | Non severe | 28 | - | - | Yes | atherosclerotic | Anticoagulant | NA | NA | NA | NA | NA | Survival |
| Li et al. ^28^ | F | NA | NA | NA | NA | severe | 18 | - | - | Yes | atherosclerotic | Anticoagulant | NA | NA | NA | NA | NA | Survival |
| Li et al. ^28^ | M | NA | smoking | None | None | severe | 35 | - | - | NA | Cardioembolic | Anticoagulant | NA | NA | NA | NA | NA | Death |
| Sierra‐Hidalgo et al. ^87^ | M | 78 | AH, DM, DLP | None | None | NA | 39 | RC | right PCA, BA occlusion | Yes | Undetermined (evaluation incomplete) | NA | 68880 | NA | NA | NA | NA | Death |
| Sierra‐Hidalgo et al. ^87^ | F | 83 | AH, DM, DLP, FA | None | None | NA | 20 | left hemianopia, facial paresis, hemiplegia, Babinski sign, hemianesthesia; right gaze deviation | right MCA occlusion | NA | Cardioembolic (AF) | NA | 2830 | NA | NA | NA | NA | Death |
| Sierra‐Hidalgo et al. ^87^ | M | 77 | AH, DM, DLP | None | None | NA | 21 | right facial paresis, hemiplegia, Babinki sign, mutism, hemianopia; left gaze deviation | left ICA-MCA occlusion | Yes | Undertermined (floating aortic thrombus) | NA | 2200 | NA | NA | NA | NA | Critically Ill |
| Sierra‐Hidalgo et al. ^87^ | M | 60 | AH, CAD | None | 1 | NA | 33 | RC, coma with unilateral decerebrate response | right and left MCA occlusion | Yes | Undetermined (evaluation incomplete) | NA | 5440 | NA | NA | NA | NA | Death |
| Sierra‐Hidalgo et al. ^87^ | M | 76 | AH, DLP | None | None | NA | 3 | left arm paresis, left arm and leg hypoesthesia | right MCA occlusion | Yes | Atherosclerotic (right cervical ICA stenosis) | NA | 810 | NA | NA | NA | NA | Survival |
| Sierra‐Hidalgo et al. ^87^ | M | 55 | None | None | None | NA | 40 | RC, right mydriasis | CTA: both VA and BA occlusion | Yes | Undertermined | NA | 850 | NA | NA | NA | NA | Critically Ill |
| Sierra‐Hidalgo et al. ^87^ | M | 61 | None | None | None | NA | 40 | RC | right and left MCA and PCA, BA occlusion | Yes | Undetermined (evaluation incomplete) | NA | 45680 | NA | NA | NA | NA | Death |
| Sierra‐Hidalgo et al. ^87^ | M | 56 | None | None | None | NA | 2 | right hemianopia, visual release hallucinations (Charles Bonnet syndrome) | Brain CT: left PCA hyperdensity; CTA: left VA occlusion | Yes | Undertermined | NA | 1030 | NA | NA | NA | NA | Survival |
| Ashrafi et al. ^88^ | F | 33 | None | None | None | lethargy | 24 | RC, global aphasia, right side hemiplegia | Brain CT: left MCA territory ischemia | Yes | Undetermined | Antiplatelet | 728 | NA | 8000 | 305000 | 24 | Death |
| Ashrafi et al. ^88^ | M | 39 | None | None | None | Cough, headache, myalgia, dyspnea | 9 | left hemiparesis, homonymous hemianopia, sensory deficit, dysarthria | Brain CT: right MCA territory ischemia | NA | NA | Antiplatelet | 810 | NA | 5500 | 183000 | 12 | Survival |
| Ashrafi et al. ^88^ | F | 49 | AH | None | None | Myalgia | 11 | left hemiplagia, heminimous hemianopia, dysarthria | Brain CT: right MCA territory ischemia | NA | NA | Antiplatelet | 954 | NA | 8700 | 146000 | 27 | Survival |
| Ashrafi et al. ^88^ | M | 40 | None | None | None | Myalgia, dyspnea | 6 | right hemiparesis, dysarthria | Brain CT: left MCA territory ischemia | NA | NA | Antiplatelet | 682 | NA | 7400 | 223000 | 5 | Survival |
| Ashrafi et al. ^88^ | M | 53 | AH | None | None | Cough, diarrhea | 5 | left hemiparesis, dysarthria | Brain CT: right MCA territory ischemia | NA | NA | Antiplatelet | 600 | NA | 4500 | 101000 | 12 | Survival |
| Ashrafi et al. ^88^ | F | 47 | AH, DM | None | None | Myalgia, cough and dyspnea | 6 | right hemiparesis, dysarthria | Brain CT: left basal ganglia ischema | NA | NA | Antiplatelet | 1293 | NA | 9000 | 210000 | 10 | Survival |
| D'Anna et al. ^89^ | F | 63 | PAD, CAD, AH | None | 1 | fever, dyspnea | 9 | neglect, dysaphasia, left arm paresis | Brain CT: Multiple and bilateral infarcts | No | Undetermined (evaluation incomplete) | Antiplatelet | 9709 | NA | 5600 | 132000 | 8,4 | Survival |
| D'Anna et al. ^89^ | M | 83 | AF | None | None | fever, dyspnea | 19 | dysphasia, right hemiplegia, sensory deficit, gaze preference, facial droop | Brain CT: Multiple infarcts; floating thrombus in the left ICA | Yes | Cardioembolic | LMWH | 1256 | NA | 5100 | 376000 | 213,4 | Critically Ill |
| D'Anna et al. ^89^ | M | 88 | None | None | None | fever, malaise, dyspnea | 3 | facial droop, right hemiparesis | Brain CT: Single infarct | NA | Cardioembolic | 14 days of Aspirin 300 mg followed by Apixaban | 20000 | NA | 27400 | 225000 | 279,2 | Survival |
| D'Anna et al. ^89^ | M | 77 | AF, stroke, COPD, AH | 1 | None | dyspnea, cough | 13 | dysarthria, dysphasia, right hemiparesis | Brain CT: Multiple and bilateral infarcts | NA | Cardioembolic | Apixaban | 3846 | NA | 7200 | 214000 | 96,2 | Survival |
| D'Anna et al. ^89^ | M | 71 | DM, smoking, AH | None | None | dyspnea | 12 | right hemianopia, right hemiparesis, dysphasia, dysarthria | Multiple infarcts with hemorrhagic transformation type PH1; floating thrombus in the left ICA | Yes | Undetermined (evaluation incomplete) | LMWH | 1557 | NA | 9500 | 590000 | 44,3 | Survival |
| D'Anna et al. ^89^ | M | 79 | DM, DLP, previous TIAs, AH | 1 | None | cough, fever | 8 | dysphasia, right arm paresis, sensory deficit | No acute infarct on first CT; 24 h MRI showed multiple and bilateral infarcts with hemorrhagic transformation type HI-1 | No | Undetermined (evaluation incomplete) | IVT + Aspirin | NA | NA | 4800 | 148000 | 18,6 | Survival |
| D'Anna et al. ^89^ | M | 70 | DM, AF, CAD | None | 1 | fever, cough | 7 | dysphasia, inattention, dysarthria | Single infarct with M1-M2 MCA occlusion | Yes | Cardioembolic | Antiplatelet | 5952 | NA | 11400 | 634000 | 80,7 | Survival |
| Cavallieri et al. ^90^ | M | 33 | None | None | None | cough, fever | NA | Headache, dysarthria, left lateral gaze palsy, retching and balance disorder | Brain CT: bilateral cerebellar ischemic lesions in the territories of the left PICA, bilateral AICA and bilateral SCA. CTA: left V1 VA occlusion up to V4 + occlusion of left PICA and AICA bilaterally | Yes | Undertermined | Fondaparinux 2.5mg/daily | 3728 | NA | NA | 466000 | 27,6 | Survival |
| Guillan et al. ^91^ | M | 67 | AH, smoking, alcohol | None | None | Cough | NA | temporo-spatial disorientation, dysarthria, partial cortical blindness and anosognosia with visual confabulation, optic ataxia, difficulty in visual scanning, simultagnosia, mild left hemihypoesthesia | Brain CT: bilateral parietooccipital and right cerebellar hypoattenuating lesions with areas of cortical hyperattenuating involvement. Brain MRI: ischaemic lesions involving the posterior segment of the right MCA, the left PCA, and a segment of the right SCA with a high signal in the long TR sequences with hematic remains of petechial cortical distribution (cortical laminar necrosis). MR angiography: no abnormalities | No | Undertermined | NA | 1777 | 1107 | 13100 | normal | 38,6 | Survival |
| Duroi et al. ^92^ | M | 74 | None | None | None | fever, cough | NA | RC | Brain CT: large infarct with hypodensity in the territory of the MCA in addition to oedema and deviation of the midline. At the transition of the left ICA to the origin of the MCA, a hyperdense artery sign was seen due to a thrombotic occlusion | Yes | Undertermined | NA | 3941 | NA | NA | normal | NA | Death |
| Doo et al. ^93^ | M | 55 | DM | None | None | asymptomatic | . | left wrist droop and sudden worsening in ED | Brain CT unremarkable; CTA: right extracranial ICA thrombus; CTP: right frontal infarction | Yes | Undetermined | EVT, eptifibatide, Aspirin | NA | NA | NA | NA | NA | Survival |
| Doo et al. ^93^ | M | 64 | smoking | None | None | cough, fever, chills, shortness of breath; in ED sudden worsening with intubation and ICU admission | NA | status epilepticus while in ICU | Brain CT/MRI: bilateral posterior hypodensities with hemorrhagic transformation; MRA did not show anbormalities. | No | Other rare causes (PRES) | NA | NA | NA | NA | NA | NA | NA |
| Kariyanna et al. ^94^ | F | 72 | obesity, DM, HLP, AH | None | None | respiratory failure | 12 | NA | Brain CT: acute infarction in left parietal lobe | NA | Cardioembolic (takotsubo) | Aspirin | 6518 | 476 | 21400 | 424000 | 270 | Death |
| Hanafi et al.^20^ | M | 65 | NA | None | None | fatigue, fever, cough | NA | RC despite discontinuation of sedation | Brain CT: multiple white matter, basal ganglia, and cerebellar hypodensities and bilateral globus pallidus hyperdensities, suggestive of hemorrhage confirmed on T2*-weighted MR imaging. Brain MRI: extensive ischemic lesions with restricted diffusion, involving the centrum semiovale, corpus callosum, basal ganglia, and cerebellum, with patchy/punctuate enhancement. MRA was normal. | No | NA | NA | NA | NA | NA | NA | NA | NA |
| Williams et al. ^95^ | F | 38 | family history of CADASIL | None | None | fever, myalgia, anosmia, ageusia | 1 | mild dysarthria | Brain CT: low attenuation within the right corona radiata; CTA: unremarkable. MR: chronic SVD pattern | No | Other rare causes (CADASIL) | aspirin | NA | NA | NA | NA | NA | Survival |
| Zhou et al. ^96^ | F | 75 | AH | None | None | cough, fatigue, shortness of breath | NA | left hemiplegia with R hemiparesis | Brain CT: bilateral cerebral infarcts involving right MCA and ACA distribution and left ACA | NA | Undetermined | Aspirin, Clopidogrel | 8000 | NA | 5600 | 119000 | 42,52 | Survival |
| TunÇ et al. ^97^ | F | 45 | DM | None | None | Fever, cough | 16 | left facial paresis, dysarthria, left hemiparesis | Brain MRI: total left MCA infarction | Yes | Undetermined | Aspirin, low dose LMWH | 803 | 264 | NA | NA | 142 | Critically Ill |
| TunÇ et al. ^97^ | F | 67 | AH | None | None | fever | 5 | dysarthria, right hemiparesis | Brain MRI: left lenticulostriate artery infarction | No | Undetermined | Aspirin, Clopidogrel | 1040 | 79 | NA | NA | 4 | Survival |
| TunÇ et al. ^97^ | M | 72 | AH | None | None | cough | 10 | RC, dysarthria, right hemiparesis | Brain MRI: infarction in the territories of the cortical branches of left MCA | Yes | Undetermined | Aspirin, low dose LMWH | 644 | 132 | NA | NA | 33 | Critically Ill |
| TunÇ et al. ^97^ | M | 77 | AH | None | None | fever, cough, shortness of breath | 2 | left hemi-hypoesthesia, mild ataxia | Brain MRI: right pontine infarction | No | Undetermined | Aspirin, Clopidogrel | 378 | 127 | NA | NA | 366 | Survival |
| Morjaria et al. ^98^ | M | 75 | CAD, CKD, DLP | None | 1 | Dyspnea | NA | Increased tone and reduced power in the lower limbs bilaterally | Brain CT: no alterations. Brain MRI: small vessel disease and infarcts in the parietal and occipital lobes, and in the midbrain and basal ganglia | No | Undetermined | NA | 8974 | 506 | 13400 | NA | 165 | Survival |
| Morjaria et al. ^98^ | M | 49 | DM, AH | None | None | cough, fever, dyspnea | NA | bilateral lower limb weakness | Brain MRI: abnormal signal in the deep white matter bilaterally, likely to represent subcortical watershed infarcts | No | small-vessel | NA | 17239 | 1848 | 29600 | NA | 279 | Survival |
| Avula et al. ^27^ | M | 73 | AH, DLP, carotid stenosis | None | None | fever, respiratory distress, altered mental status | NA | altered mental status | Brain CT: large acute infarct of the left MCA territory with hyperdense appearance of left MCA consistent with an acute thrombus | Yes | Undertermined | Aspirin | NA | NA | 12320 | 182000 | 26,22 | Death |
| Avula et al. ^27^ | F | 83 | AH, DLP, DM | None | None | fever | 2 | left facial droop, slurred speech | Brain CT: hypodensity in the right frontal lobe representing acute infarction. CTA: focal moderate stenosis of right MCA | No | Undertermined | Supportive treatment | NA | NA | 4950 | 138000 | NA | Death |
| Avula et al. ^27^ | F | 80 | AH | None | None | asymptomatic | 36 | Left hemiplegia and aphasia | Brain CT: acute right MCA stroke. CTA: occlusion of the right ICA at origin. CTP: 305 cc core infarct in the right MCA distribution and a surrounding 109 cc ischemic penumbra | Yes | Undertermined | Supportive treatment | 13966 | 891 | 18890 | 380000 | 16,24 | Death |
| Avula et al. ^27^ | F | 88 | AH, CKD, DLP | None | None | shortness of breath, cough | NA | transient 15-minute episode of right arm weakness and numbness along with word-finding difficulty | Brain MRI: acute infarct in the left medial temporal lobe. MRA: mild stenosis of the right M1 MCA segment | Yes | Undetermined | Aspirin | 3442 | 135 | 7500 | 176000 | 12,7 | Survival |
| Gunasekaran et al. ^99^ | F | 40 | None | None | None | cough, fever, shortness of breath | NA | RC sluggish pupils and absent corneal responses despite discontinuation of sedation | Brain CT: large right MCA territory infarct with extensive mass effect, including midline shift and downward herniation | Yes | Undetermined | Supportive treatment | 2807 | 3079 | 14000 | 303000 | NA | Death |
| Deliwala et al.^100^ | F | 31 | None | None | None | fever, rhinorrhea, cough, myalgias, vomiting congestion, abdominal cramping | NA | Confusion, left-hand paralysis | Brain CT: hypodensity in the right MCA territory | NA | Undertermined | Aspirin | 3640 | 437 | 9900 | 173000 | 38,09 | Survival |
| Salahuddin et al. ^101^ | M | 75 | NA | NA | NA | respiratory symptoms | 12 | right gaze deviation and left arm drift | Brain CT and CTA: right M3 MCA branch occlusion with matching infarct | No | Undertermined | Supportive treatment | NA | 393 | NA | NA | 27 | Death |
| Zhai et al. ^102^ | M | 79 | None | None | None | cough | NA | right limb weakness, runs of speech were not fluent enough with tongue deviation. | Brain CT: left hemisphere subcortical infarction | No | Cardioembolic (AF) | Clopidogrel | NA | NA | 7080 | NA | 36,1 | Survival |
| Co et al. ^103^ | F | 62 | AH, DM, DLP, previous TIA | 1 | None | cough, colds, shortness of breath | 4 | severe dysarthria and right upper and lower extremity weakness | Brain CT: hypodensity in the left centrum semiovale and corona radiata. CTA: significant stenosis in the left M1 MCA | Yes | atherosclerotic | IVT + Aspirin | 1160 | 4609 | 13200 | 409000 | 192 | NA |
| Rudilosso et al. ^104^ | M | 50 | None | None | None | bilateral pneumonia | NA | right facial palsy with mild ipsilateral limb weakness | CTP: small focal hypoperfusion in the paramedian perforating vascular territory supplying the left medial thalamus. Brain MRI study acquired 48 hours later: other 2 punctate acute ischemic lesions in each cerebellar hemisphere | No | Undertermined | Heparin infusion | NA | NA | NA | NA | NA | Survival |
| Papi et al. ^105^ | F | 79 | AF, AH, CAD, DM | None | 1 | asymptomatic | 24 | NA | Brain CT, CTA, CTP: left MCA occlusion, large area of ischemic penumbra | Yes | Undertermined | EVT | NA | NA | NA | NA | NA | Death |
| Yeboah et al. ^106^ | F | 49 | DM, AH | None | None | shortness of breath, fever, fatigue | 14 | weakness in the left limbs, sensory neglect, left hemianopsia and right gaze deviation | CTA: thrombus in the right MCA and a filling defect in the left carotid bulb. CTP: mismatch between cerebral blood volume and mean transit time in the territory of the right MCA | Yes | Undertermined | IVT + EVT (TICI: 3) | NA | 3578 | NA | NA | 187 | Survival |

**Abbreviations:** ACA: Anterior Cerebral Artery; AF: Atrial Fibrillation; AH: arterial hypertension; AICA: anterior inferior cerebellar artery; ARDS: acute respiratory distress syndrome; BA: basilar artery; CAD: coronary artery disease; CCA: common carotid artery; CHF: Congestive heart failure; CKD: chronic kidney disease; COPD: Chronic obstructive pulmonary disease; CVD: cerebrovascular disease; CT: Computed Tomography; CTA: computed tomography angiography; CTP: CT perfusion; DLP: dyslipidemia ; DM: diabetes mellitus type II; EVT: endovascular treatment; F: female; FLAIR: fluid attenuated inversion recovery; ICA: internal carotid artery; ICH: Intracerebral hemorrhage; IVT: intravenous thrombolysis; LMWH: low molecular weight heparin; M: male; MCA: middle cerebral artery; MRA: magnetic resonance angiography; MRI: Magnetic Resonance Imaging; MRS: Modified Rankin Scale; NIHSS: National Institutes of Health Stroke Scale; PAD: peripheral arterial disease; PCA: Posterior Cerebral Artery; PComm: posterior communicating artery; PICA: Posterior-Inferior Cerebellar Artery; RC: reduced consciousness; SAS: Sleep Apnea Syndrome; SWI: Susceptibility-Weighted Imaging; VA: Vertebral Artery.
