# Supplementary material for: COVID-19 and Cerebrovascular Diseases: A Systematic Review and Perspectives for Stroke Management
Source: Front Neurol. 2020 Nov 5;11:574694. doi: 10.3389/fneur.2020.574694 (PMC7674955; doi:10.3389/fneur.2020.574694)
Supplement: Supplementary file 2 [file Table_2.DOCX]

| **Author** | **Sex** | **Age** | **CVD Risk Factors** | **COVID symptoms** | **NIHSS admission** | **Neurological manifestation** | **BRAIN MRI/CT abnormalities** | **Type of intracranial bleeding** | **Concomitant treatment at the moment of SAH/ICH** | **D-Dimer (ng/mL)** | **Ferritin (ng/mL)** | **WBC counts (/mm3)** | **Platelet count (/mm3)** | **CRP (mg/L)** | **Outcomes** |
| --- | --- | --- | --- | --- | --- | --- | --- | --- | --- | --- | --- | --- | --- | --- | --- |
| Al Saiegh et al ^25^ | M | 31 | None | Malaise, fever, cough, arthralgia | NA | RC and headache | Brain CT: SAH in the posterior cranial fossa, including the fourth ventricle. DSA: right ruptured dissecting PICA aneurysm | SAH | Flow-diverting stent | NA | NA | NA | NA | NA | Survival |
| Morassi et al. ^64^ | M | 57 | AH, thrombocytosis | fever, cough | NA | RC, Bilaterally fixed and dilated pupils and a GCS of 3. | Brain CT: hemorrhages in both cerebellar hemispheres (diameter of 4.8 cm on the right one, and 3.6 cm on the left one) with compression of the fourth ventricle and the brainstem | ICH | LMWH | 2866 | NA | NA | NA | 214 | Death |
| Morassi et al. ^64^ | M | 57 | None | fever, cough | NA | RC, Bilaterally fixed and dilated pupils and a GCS of 3. | Brain CT: diffuse cerebral edema with a large right frontal ICH (6.0 × 5.2 cm on the axial plane) extending to the ventricular system. Four additional smaller intra-axial hemorrhages occurred in both hemispheres | ICH | LMWH | within normal values | NA | NA | NA | 21 | Death |
| Li et al.^28^ | M | 62 | smoking, alcohol | severe form | NA | NA | Brain CT: left basal ganglia ICH | ICH | NA | NA | NA | NA | NA | NA | Death |
| Radmanesh et al. ^107^ | M | 74 | heparin treatment | patient intubated due to hypoxic respiratory failure | NA | RC | Brain CT: extensive supra- and infratentorial acute hemorrhage with subarachnoid and intraventricular extensions, along with cerebral swelling and hypodensity (likely hypoperfusion injury), as well as uncal, subfalcine, and transtentorial herniations | ICH | NA | NA | NA | NA | NA | NA | NA |
| Radmanesh et al. ^107^ | F | 61 | compensated hepatic cirrhosis (due to primary sclerosing cholangitis) | patient hospitalized in the intensive care unit | NA | R weakness and numbness | Brain CT: left parietal ICH with surrounding vasogenic edema. | ICH | NA | NA | NA | NA | NA | NA | NA |
| Sharifi-Razavi et al. ^108^ | M | 79 | None | fever, cough | NA | RC, bilateral extensor plantar reflexes | Brain CT: massive ICH in the right hemisphere with intraventricular bleeding and SAH | ICH | NA | NA | NA | 590 | 210000 | 10 | NA |
| Zulfiqar et al. ^109^ | F | 65 | AH, autoimmune hypothyroidism, immune thrombocytopenia | fatigue, fever, dry cough, and abdominal discomfort | NA | Frontal headache | Brain CT: SAH in the right frontal lobe. | SAH | LMWH | NA | NA | NA | 1000 | 55 | NA |
| Hussain et al. ^110^ | F | 69 | tissue aortic valve replacement in 2012; CHF | At the beginning cough, fever, shortness of breath. At time of ICH: the pneumonia got significantly worse with multiorgan failure including severe acute kidney injury requiring heparin for renal replacement therapy | NA | acute dilated L pupil | Brain CT: large acute left ICH with midline shift | ICH | LMWH | NA | NA | NA | NA | NA | Death |
| Muhammad et al. ^111^ | F | 60 | None | none | NA | RC, coma | left frontal ICH with ventricle bleeding from a ruptured pericallosal artery aneurysm around 5 mm in size | ICH + SAH | None | NA | NA | 14200 | NA | 11 | Survival (Rehab) |
| Vu et al. ^112^ | M | 30 | None | none | NA | dysarthria, R hemiparesis, and a R facial droop | Brain CT: 1.7-cm acute left basal ganglia ICH | ICH | None | NA | NA | NA | NA | NA | NA |
| Carroll et al. ^113^ | M | 62 | history of colon cancer | fever, shortness of breath, fatigue, cough | NA | RC, coma and absent brainstem reflexes with the exception of the vestibulocochlear reflex | Brain CT: multifocal ICH with intraventricular extension, mass effect, and evidence of global anoxic injury, cerebral edema, and downward herniation of the cerebellar tonsils | ICH | heparin infusion | 2997 | 3072 | 7750 | 170000 | 165 | Death |
| Carroll et al. ^113^ | M | 74 | history of tongue cancer, AH, carotid stenosis status post right carotid stent | dyspnea | NA | RC, persistent coma with no brainstem reflexes | Brain CT: large ICH in the right temporal, occipital, and parietal lobes with intraventricular extension, as well as a large brainstem hemorrhage | ICH | heparin infusion and aspirin 81 mg daily | 2887 | 8530 | 9700 | 385000 | 232 | Death |
| Al-olama et al. ^114^ | M | 36 | None | fever, headache, body pain, cough, diarrhea and vomiting | NA | drowsiness and confusion | Brain CT: R frontal ICH associated with SAH in the ipsilateral sylvian fissure and frontal and temporal lobes and subdural hematomas | meningoencephalitis with SAH, ICH and subdural hematomas | None | 790 | NA | 12900 | NA | normal | NA |
| Reddy et al. ^71^ | M | 48 | AH | NA | 10 | left hemiparesis | CT: Dorsolateral pontine hemorrhage | ICH | NA | 2300 | NA | 13600 | 174000 | NA | Survival |
| Franceschi et al. ^73^ | M | 37 | AH, obesity | Fever, dyspnea | NA | NA | Brain CT: ICH in the cerebellar hemispheres and diffuse cerebral edema | ICH | NA | elevated D-dimer | elevated Ferritin | NA | NA | NA | Death |
| Rustemi et al. ^115^ | F | 68 | None | fever | NA | Headache | CTA: SAH from the 12x5mm Pcom aneurysm | SAH | Coiling of aneurysm | 790 | NA | NA | NA | NA | Survival |
| Agarwal et al. ^116^ | M | 56 | AH | fever, cough | NA | sudden onset left hemiparesis and loss of consciousness | Brain CT: right pontine ICH with intraventricular extension | ICH | NA | NA | NA | 23320 | 220000 | 41 | Critically Ill |
| Agarwal et al. ^116^ | M | 72 | DM | None | NA | L weaknes | Brain CT: right frontal lobar ICH with intraventricular extension | ICH | NA | NA | NA | 11260 | 165000 | NA | Survival |
| Kim et al. ^117^ | F | 53 | None | None | NA | Dysarthria and left hemiparesis | Brain CT: right external capsule and putamen ICH with the ipsilateral intraventricular extension | ICH | NA | 410 | NA | NA | 141000 | NA | Survival (Rehab) |
| Krett et al. ^118^ | M | 69 | CAD, AH, DM | cough, fatigue followed by respiratory distress | NA | RC, unresponsive and diffusely paretic without sedation. | Brain CT at admission: negative; Brain MRI 13 days later: multicompartmental hemorrhages with mild surrounding vasogenic edema and no abnormal enhancement. CT angiogram: no underlying vasculopathy | ICH | NA | NA | NA | NA | normal | NA | Survival (Rehab) |
| Hernández-Fernández et al.^16^ | F | 51 | AH, smoking | none | NA | aphasia, right hemiplegia and RC (coma) | Brain CT: extensive left basal ganglia ICH with ventricular extension, SAH | ICH + SAH | NA | 648 | NA | NA | 402000 | NA | Death |
| Hernández-Fernández et al. ^16^ | M | 69 | AH | fever, progressive dyspnoea | NA | RC, difficulty arousing him after tracheal extubation | Brain CT: left frontal lobar ICH | ICH | NA | 6566 | 1460 | NA | 178000 | 4,5 | Survival |
| Hernández-Fernández et al. ^16^ | M | 61 | None | ARDS | NA | adult onset focal seizures | Brain CT: left temporal ICH with associated SAH | ICH + SAH | NA | 4594 | 657 | NA | 268000 | 36 | Death |
| Hernández-Fernández et al. ^16^ | M | 64 | AH, DM, CAD | fever, dyspnoea and severe bilateral interstitial pneumonia | NA | RC difficulty arousing him after tracheal extubation | Brain CT: focal cortico-subcortical ICH, and multiple foci of associated SAH. | ICH + SAH | NA | 3546 | 1463 | NA | 129000 | 1,9 | Survival |
| Hernández-Fernández et al. ^16^ | M | 68 | AH, DLP, DM, SAS | fever, dyspnoea and severe bilateral interstitial pneumonia | NA | RC, difficulty arousing him after tracheal extubation | Brain CT: small left frontal lobar ICH and SAH | ICH + SAH | NA | 1585 | 2637 | NA | 78000 | 1,2 | Survival |
| Ghani et al.^119^ | M | 59 | AH | acute hypoxic respiratory failure | NA | RC, absent brainstem reflexes | Brain CT: SAH and ICHs within the posterior fossa | ICH+SAH | unfractionated heparin | 3000 | NA | NA | NA | NA | Death |
| Ghani et al. ^119^ | F | 61 | DM | acute hypoxic respiratory failure | NA | RC, fixed dilated pupils with no brainstem reflexes | Brain CT: diffuse brain anoxia with scattered SAHs, a subdural hematoma with midline shift and tonsillar herniation | SAH | apixaban switched to unfractionated heparin | NA | NA | NA | NA | NA | Death |
| Ghani et al. ^119^ | F | 59 | AH | acute hypoxic respiratory failure | NA | RC, comatose despite being off intravenous sedation for 1 week | Brain CT: large ICH and cerebral edema, with transtentorial-herniation causing brainstem compression | ICH | apixaban switched to enoxaparin | NA | NA | NA | NA | NA | Death |
| Wee et al. ^120^ | M | 64 | NA | acute hypoxic respiratory failure | NA | RC, dilated and unresponsive pupils | Brain CT: bihemispheric multifocal ICH, with multiple fluid-blood levels | ICH | ECMO + protocolized anticoagulation therapy | NA | NA | NA | NA | NA | NA |
| Benger et al. ^121^ | M | 41 | AH, DM | cough, fatigue and fever | NA | right hemiplegia | Brain CT: left frontal ICH requiring craniotomy and evacuation | ICH | LMWH | 1920 | NA | 20300 | 510000 | 1 | Critically Ill |
| Benger et al. ^121^ | F | 54 | AH, DM | cough | NA | dysarthria and left hemiparesis | Brain CT: right frontal lobe ICH; CTA was normal | ICH | Warfarin | 1400 | NA | 6100 | 270000 | 7 | Critically Ill |
| Benger et al. ^121^ | M | 50 | AH | cough, pleuritic chest pain and shortness of breath | NA | NA | Brain CT: right frontal lobe ICH. Intracranial CTA: normal. | ICH | LMWH | 8961 | NA | 15200 | 72000 | 138 | Critically Ill |
| Benger et al. ^121^ | F | 64 | None | cough, shortness of breath and fever | NA | RC, low GCS post- sedation wean | Brain CT: subacute right gangliocapsular ICH; CTA was normal | ICH | Intravenous heparin | 8000 | NA | 15800 | 221000 | 330 | Critically Ill |
| Benger et al. ^121^ | M | 52 | AH, DLP, CAD | shortness of breath, cough, fevers and pleuritic chest pain | NA | RC, low GCS post- sedation wean | Brain CT: subacute ICH in the right perirolandic region, with smaller petechial haemorrhages in the left paramedian frontal lobe and left cingulate sulcus. CTA: normal | ICH | Intravenous heparin | 7580 | NA | 14600 | 313000 | 77 | Critically Ill |

**Abbreviations**: ACA: Anterior Cerebral Artery; AF: Atrial Fibrillation; AH: arterial hypertension; AICA: anterior inferior cerebellar artery; ARDS: acute respiratory distress syndrome; BA: basilar artery; CAD: coronary artery disease; CCA: common carotid artery; CHF: Congestive heart failure; CKD: chronic kidney disease; COPD: Chronic obstructive pulmonary disease; CVD: cerebrovascular disease; CT: Computed Tomography; CTA: Computed tomography angiography; CTP: CT perfusion; DLP: dyslipidemia ; DM: diabetes mellitus type II; F: female; FLAIR: fluid attenuated inversion recovery; ICA: internal carotid artery; ICH: Intracerebral hemorrhage; LMWH: low molecular weight heparin; M: male; MCA: middle cerebral artery; MRA: magnetic resonance angiography; MRI: Magnetic Resonance Imaging; MRS: Modified Rankin Scale; NIHSS: National Institutes of Health Stroke Scale; PAD: peripheral arterial disease; PCA: Posterior Cerebral Artery; PComm: posterior communicating artery; PICA: Posterior-Inferior Cerebellar Artery; RC: reduced consciousness; SAS: Sleep Apnea Syndrome; SAH: Subarachnoid hemorrhage; SWI: Susceptibility-Weighted Imaging; VA: Vertebral Artery.
