# Supplementary material for: COVID-19 and Cerebrovascular Diseases: A Systematic Review and Perspectives for Stroke Management
Source: Front Neurol. 2020 Nov 5;11:574694. doi: 10.3389/fneur.2020.574694 (PMC7674955; doi:10.3389/fneur.2020.574694)
Supplement: Supplementary file 3 [file Table_3.DOCX]

| **Author** | **Sex** | **Age** | **CVD Risk Factors** | **COVID symptoms** | **NIHSS admission** | **Neurological manifestation** | **BRAIN MRI/CT abnormalities** | **Mechanism of stroke** | **Treatment of CVST** | **D-Dimer (ng/mL)** | **Ferritin (ng/mL)** | **WBC counts (/mm3)** | **Platelet count (/mm3)** | **CRP (mg/L)** | **Outcomes** |
| --- | --- | --- | --- | --- | --- | --- | --- | --- | --- | --- | --- | --- | --- | --- | --- |
| Li et al. ^28^ | M | 32 | DM, smoking, drinking | NA | NA | NA | NA | CVST | Anticoagulant | NA | NA | NA | NA | NA | Survival |
| Hughes et al. ^122^ | M | 59 | DM, AH, obesity | none | 10 | right fronto-temporal headache, followed by reduced power and numbness in right limbs, slurred speech and expressive dysphasia | CT scan: hyperdensity within the SSS, right TS, SS and upper right internal jugular vein. CTA: filling defect in the right TS and SS involving the torcular | CVST | LMWH | NA | NA | 6300 | 234000 | 20 | Survival |
| Cavalcanti et al. ^123^ | M | 38 | none | vomiting, diarrhea | 14 | headache, altered mental status followed by extensor posturing of the arms and clonus | CT scan: hyperdensity in the straight sinus, distal SSS, torcular and right TS. CT venography: CVST including a near-occlusive thrombus in the right internal cerebral vein | CVST | Enoxaparin 70 mg was given subcutaneously twice a day followed by percutaneous VME | NA | NA | 16710 | 141000 | NA | Death |
| Cavalcanti et al. ^123^ | F | 41 | none | NA | NA | confusion, aphasia followed by RC | CT scan: venous infarction in the left basal ganglia, thalamus and mesial temporal lobe with hemorrhagic transformation, intraventricular hemorrhage and obstructive hydrocephalus | CVST | Heparin infusion | 2032 | NA | 9950 | 239000 | NA | Death |
| Cavalcanti et al. ^123^ | M | 23 | none | body aches, fever, dry cough | NA | headache and RC | Brain MRI: confluent, non-enhancing regions of reduced diffusion throughout the subcortical and deep hemispheric white matter bilaterally. Punctate foci of susceptibility artifact consistent with petechial hemorrhage on GRE images. | CVST | NA | 18431 | NA | 8840 | 86000 | NA | Death |
| Poillon et al. ^124^ | F | 62 | obesity | fever, cough, dyspnea | NA | headache and altered vision, rapidly followed by R hemicorporeal deficit and RC | Brain CT and brain MRI: large confluent intrapenchymal hemorrhage in left frontal and temporal lobes. CTA: CVST of left TS, straight vein, vein of Galen and internal cerebral veins | CVST | NA | 1420 | NA | 20220 | NA | NA | NA |
| Poillon et al. ^124^ | F | 54 | History of breast cancer | fever, asthenia | NA | severe headache | Brain CT and brain MRI: large hemorrhagic infarction in the left temporal lobe. CTA and MRA: CVST of the left TS. | CVST | NA | 2360 | NA | 18320 | NA | 170,8 | NA |
| Klein et al. ^125^ | F | 29 | - | cough, low grade fever, mild shortness of breath, mild headache | NA | mild headache, generalized tonic-clonic seizures, RC, global aphasia, decreased blink to threat on the R and a mild to moderate R facial palsy. | CT scan: left temporo-parietal hemorrhagic venous infarction with edema and mass effect and CVT in left distal TS and SS. Brain MRI: hyperintense DWI signal of the left temporo-parietal hemorrhagic infarction with mass effect. MRA: absence of flow in the left TS and SS and left internal jugular vein | CVST | acetazolamide 500 mg twice daily | 2876 | 1040 | 8760 | 335000 | 111.74 | Survival |
| Garaci et al. ^126^ | F | 44 | none | fever, cough, dyspnea | NA | headache, altered mental status, aphasia and right hemiparesis | CTA: empty delta sign in the vein of Galen, straight sinus and in the torcular herophili with poor rappresentation of left internal cerebral vein. | CVST | NA | 5975 | NA | 9600 | 42000 | NA | NA |
| Roy-Gash et al. ^127^ | F | 63 | - | Fever, cough, anosmia | NA | Aphasia and right hemiplegia | CTA: extensive venous thrombosis. Located in the straight sinus and left TS. Voluminous left temporal hemorrhage with venous thrombosis. | CVST | Heparin infusion | NA | 1427 | NA | NA | NA | Death |
| Baudar et al. ^128^ | F | 33 | Obesity | Myalgia, Cough and Fever, Anosmia, Dyspnea, dysgeusia | NA | Headache | brain MRI: L parietal cortical CVT | CVST | Dabigatran | 902 | NA | NA | NA | NA | Survival |
| Rigamonti et al. ^129^ | M | 54 | None | Cough, fatigue, fever | NA | Headache and aphasia | Brain CT: ischaemic hypodensity involving left basal ganglia and thalamo-capsular region with a small haemorrhagic hyperdensity in the context of nucleus caudatus, associated with mass effect and shift of midline structure. CTA: thrombosis involving deep veins of the left hemisphere. | CVST | LMWH | 3000 | NA | NA | NA | 97 | Death |
| Hemasian et al. ^130^ | M | 65 | none | NA | NA | RC, upward gaze and tongue biting | Brain MRI: hemorrhagic infarct in right temporal lobe and right SS and TS thrombosis | CVST | Anticoagulant therapy | NA | NA | NA | NA | NA | Survival |
| Chougar et al. ^131^ | M | 72 | none | mild respiratory symptoms | NA | sudden left hemiparesis, altered mental status, and refractory status epilepticus | Brain CT: right deep hemispheric hypodensity with some hyperdense areas, involving the thalamus, basal ganglia, internal capsule and splenium of the corpus callosum, and the deep white matter; CTV: internal cerebral veins and the vein of Galen thrombosis | CVST | NA | NA | NA | NA | NA | NA | Death |

**Abbreviations**: ACA: Anterior Cerebral Artery; AF: Atrial Fibrillation; AH: arterial hypertension; AICA: anterior inferior cerebellar artery; ARDS: acute respiratory distress syndrome; BA: basilar artery; CAD: coronary artery disease; CCA: common carotid artery; CHF: Congestive heart failure; CKD: chronic kidney disease; COPD: Chronic obstructive pulmonary disease; CVD: cerebrovascular disease; CVT: cerebral venous thrombosis; CT: Computed Tomography; CTA: Computed tomography angiography; CTP: CT perfusion; DLP: dyslipidemia ; DM: diabetes mellitus type II; EVT: endovascular treatment; F: female; FLAIR: fluid attenuated inversion recovery; ICA: internal carotid artery; ICH: Intracerebral hemorrhage; IVT: intravenous thrombolysis; Hypertension: HP; L: left; LMWH: low molecular weight heparin; M: male; MCA: middle cerebral artery; MRA: magnetic resonance angiography; MRI: Magnetic Resonance Imaging; MRS: Modified Rankin Scale; NIHSS: National Institutes of Health Stroke Scale; PAD: peripheral arterial disease; PCA: Posterior Cerebral Artery; PComm: posterior communicating artery; PICA: Posterior-Inferior Cerebellar Artery; R: right; RC: reduced consciousness; SAS: Sleep Apnea Syndrome; SAH: Subarachnoid hemorrhage; SS: sigmoid sinus; SSS: superior sagittal sinus; SWI: Susceptibility-Weighted Imaging; TS: transverse sinus; VA: Vertebral Artery; VMT: venous mechanical thrombectomy.
